# Huber Theorem Revisited in Dimension 4

**DOI:** 10.1007/s12220-026-02470-5

**Published:** 2026-05-22

**Authors:** Paul Laurain, Dorian Martino

**Affiliations:** 1 Departement de Mathématiques, Université Gustave Eiffel, Paris, France; 2 Department of Mathematics, ETH Zürich, Rämistrasse 101, 8092 Zürich, Switzerland

**Keywords:** Conformal metric, Bach tensor, Coulomb gauge, ε-regularity, 35J48, 35J70, 30F45, 53C18, 58J90

## Abstract

We study the second Huber theorem in dimension 4. Given a metric having a pointwise singularity with $L^p$-bounds on the Bach tensor, we construct a conformal metric which is regular across the singularity. To do so, we introduce another Coulomb-type condition, similar to the case of Yang–Mills connections. This enables us to obtain a conformal metric satisfying an ε-regularity property. We obtain a generalization of the two-dimensional case that can be applied to study the singularities of Bach-flat metrics and immersions with second fundamental forms in $W^{2,\frac{4}{3}+\varepsilon }$.

## Introduction

Isolated singularities of manifolds arise frequently in geometric variational problems, for instance in the study of harmonic maps and their generalizations [1, 26, 43, 44], Willmore surfaces [10, 45, 50], but also Einstein and Bach-flat metrics [5, 6, 59, 60] or Yang–Mills connections [55]. In such problems, one usually have an a priori estimate taking the form of an $L^p$ bound on some curvature quantity (such as the second fundamental form for "extrinsic" problems or the Riemann tensor for "intrinsic" problems). A natural question is to understand the geometric behaviour across the singularity: is it possible to find a suitable extension across the singularity? In 1958, Huber proved, in his seminal paper [40], the following result on the topology of surfaces with finite total negative curvature.

### Theorem 1.1

Let (*M*, *g*) be an orientable Riemannian surface of class $C^2$, open and complete such that

$$ \int _M K^-_g \, d\textrm{vol}_g <+\infty , $$

where $K^-_g {:}{=}\max (0,-K_g)$ and $K_g$ is the Gauss curvature. Then *M* has finite topological type and moreover

$$ \int _M \vert K_g\vert \, d\textrm{vol}_g <+\infty . $$

Here finite topological type means that there exists a closed (i.e. compact and without boundary) surface $\overline{M}$ and a finite set of points $p_1,\dots ,p_N \in \overline{M}$ such that *M* is homeomorphic to $\overline{M}\setminus \{p_1,\dots ,p_N\}$. Once the topological type is fixed, a natural question concerns the conformal type, i.e. which kind of Riemann structure is supported by such surfaces. Thanks to Hopf–Rinow theorem, (*M*, *g*) is complete if every divergent path on *M* has infinite length. In the same paper [40], Huber also describes the conformal class of (*M*, *g*) in Theorem 1.1 by proving the following result.

### Theorem 1.2

Let (*M*, *g*) be an orientable surface of class $C^2$, open and complete such that

$$ \int _M K^-_g \, dv_g <+\infty . $$

Then *M* has finite conformal type.

Here finite conformal type means that there exists a closed Riemann surface $\overline{M}$ and a finite set of points $p_1,\dots ,p_N \in \overline{M}$ such that *M* is conformally equivalent to $\overline{M}\setminus \{p_1,\dots ,p_N\}$. In other words, there exist a $C^1$-diffeomorphism $\psi :M \rightarrow \overline{M}\setminus \{p_1,\dots ,p_N\}$ and a metric $g_0$ on $\overline{M}$ having constant Gaussian curvature, such that $\psi ^*(g_0)=e^{2u}g$ for some continuous function $u:M\rightarrow \mathbb {R}$.

In 1995, inspired by the work of Toro [61], Müller and Šverák [52] proved an extrinsic version of the Huber theorem: assuming for initial regularity only the fact that the second fundamental form lies in $L^2$, they proved that those surfaces admit a Lipschitz conformal parametrization. Around a potential singularity, they prove the following theorem, where $\mathbb {D}$ is the unit disk centred at the origin in $\mathbb {C}$.

### Theorem 1.3

([52], Lemma A.5 in [54]) Let $\Phi :\mathbb {D}\setminus \{0\} \rightarrow \mathbb {R}^m$ be a conformal immersion such that $\Phi \in W^{1,2}( \mathbb {D}\setminus \{0\})$ and its Gauss map is also in $W^{1,2}( \mathbb {D}\setminus \{0\})$. Then there exists an integer $\theta _0\ge 1$ such that, up to translation, the following holds for some constant C>1 in a neighbourhood of the origin

$$ C^{-1}\, |z|^{\theta _0} \le \vert \Phi (z)\vert \le C\, \vert z\vert ^{\theta _0} \qquad \text { and } \qquad C^{-1}\, |z|^{\theta _0-1} \le \vert \nabla \Phi (z)\vert \le C\, \vert z\vert ^{\theta _0-1}. $$

Moreover, if we denote the conformal factor

$$ \lambda (z) = \frac{1}{2}\log \left( \frac{1}{2} \vert \nabla \Phi (z)\vert ^2 \right) = (\theta _0-1)\log (\vert z\vert )+ u(z), $$

then the remainder *u* lies in $W^{2,1}(\mathbb {D})$ and we have

$$\begin{aligned} {\left\{ \begin{array}{ll} \nabla \lambda \in L^{2}(\mathbb {D}) & \text { if } \theta _0=1,\\ \nabla \lambda \in L^{(2,\infty )}(\mathbb {D}) & \text { if } \theta _0\ge 2. \end{array}\right. } \end{aligned}$$

Here $L^{(2,\infty )}$ denotes the weak-$L^2$ space also known as Lorentz space, see [34, Section 1.4] for a precise definition. The key ingredient in the proof of Theorem 1.3 is the fact that the Gauss curvature is the Jacobian of the gradient of the Gauss map. Hence the Gauss curvature lies in a strictly smaller space than $L^1$, namely in the Hardy space $\mathcal {H}^1$, see the seminal work of Coiffman, Lions, Meyer and Semmes [24]. Thus, Theorem 1.3 follows by combining the $\mathcal {H}^1$-$\textrm{BMO}$ duality and the Liouville equation.

The main goal of this paper is to provide possible generalizations to Theorem 1.2 and Theorem 1.3 in higher dimensions and more specifically in the 4-dimensional setting. That is to say, given a metric *g* of class $C^{\infty }$ on $\mathbb {B}^4\setminus \{0\}$, where $\mathbb {B}^4$ stands for the unit 4-dimensional Euclidean ball centred at the origin^1^, under which condition can we find a metric *h* in the conformal class of *g* that can be extended through the singularity? This question has already been addressed by various authors, who focused mainly on the generalization of Theorem 1.1, namely the topological part. The first result in this direction has been obtained in 2002 by Chang, Qing and Yang for the conformally flat case.

### Theorem 1.4

(Theorem 2 of [20]) Let $(\Omega \subset S^4, g=e^{2w}g_0)$ be a complete conformally flat metric satisfying:

(i) There exists $0<c_1<c_2$ such that scalar curvature $\textrm{Scal}_g$ verifies $c_1 \le \textrm{Scal}_g \le c_2$ and $|\nabla \textrm{Scal}_g|_g \le c_2$,
(ii) The Ricci curvature has a lower bound,
(iii) The *Q*-curvature is integrable, i.e.
  $$\begin{aligned} \int _\Omega \vert Q_g\vert \, d\textrm{vol}_g <+\infty . \end{aligned}$$

Then it holds $\Omega =S^4\setminus \{p_1,\dots ,p_k\}$.

Independently, Carron and Herzlich [18] proved in 2002 that an open set Ω of a compact manifold $(M^n,g_0)$ (for n>2) endowed with a complete conformal metric $g=e^{2u}g_0$ is also given by $\Omega =M\setminus \{p_1,\ldots ,p_k\}$, under some conditions on the volume growth of balls for *g* and the finiteness of the $L^{\frac{n}{2}}$-norm of the Ricci tensor of *g*. This result has later been improved by Carron in 2020 [17, Corollary C]. Finally in 2022, Chen and Li reached the following optimal version.

### Theorem 1.5

(Theorem 1.4 in [22]) Let Ω an open set of $(M^n,g_0)$ a Riemannian manifold of dimension n>2. Assume that Ω is endowed with a complete Riemannian metric *g* which is conformal to $g_0|_{\Omega }$ and such that:

$$\begin{aligned} \int _\Omega \vert \textrm{Ric}\vert ^{\frac{n}{2}}\, d\textrm{vol}_g < +\infty . \end{aligned}$$

Then $M\setminus \Omega$ is discrete. In particular, if $(M,g_0)$ is compact then it holds $\Omega =M\setminus \{p_1,\dots ,p_J\}$ for some points $p_1,\ldots ,p_J\in M$.

Theorem 1.4 and Theorem 1.5 are in the spirit of the first Huber theorem, i.e. controlling the topology of the domain. But it is important to notice that those results are not a complete generalization of Theorem 1.1, since they need *g* to be a priori conformally equivalent to a background metric $g_0$ which extends smoothly outside of the domain of definition of *g*. The content of Theorem 1.2 is to prove, in dimension 2, that such an extension exists. If one would like a strict equivalent of Huber’s theorem, one should also prove the existence of such an extension. In 1997, Herzlich [38] studied the conformal compactification of asymptotically flat Riemannian manifolds $(M^n,g)$ of dimension n≥3 and proved the following result, see Section 3 for the definitions.

### Theorem 1.6

(Theorem C in [38]) Any asymptotically flat Riemannian manifold, where the Weyl curvature tensor decays as $r^{-2-\epsilon }$ at infinity and the Cotton-York tensor decays as $r^{-3-\epsilon }$ (with ϵ>0), is obtained via stereographic projection from some compact $C^0$-class Riemannian manifold.

The main idea of the proof of the above result is to study the conformal connections introduced by Cartan [19] in 1924. He proves that the conformal fiber bundle does not degenerate at infinity, and is actually a Hölder-continuous bundle. The underlying analysis is based on the study of elliptic equations on weighted Sobolev spaces. Considering an inversion, this theorem can be translated to give an extension to a metric defined on $\mathbb {B}\setminus \{0\}$ and having finite volume. For a more recent result for compactification in dimension 3, see also [7].

Our main motivation is to develop a similar result that would be applicable to variational problems such as the study of compactness properties of Bach-flat metrics or generalizations of Willmore surfaces in dimension 4. In these questions, the setting is usually a sequence of manifolds which converges strongly everywhere except at a finite number of points and one would like to understand the behaviour of these manifolds across these singularities. This is the so-called bubbling convergence which usually follows from an ε-regularity property. For 2-dimensional problems see for instance the works [10, 44, 47] and the references therein. In dimension 4, see for instance in the study of Einstein metrics or Bach-flat metrics, Anderson [5, 6] and Tian–Viaclovksy [59, 60] proved that the only possible singularities occurring along a sequence of bounded energy are orbifold singularities, see also [12].

Our main contribution is to change the nature of the assumptions of Theorem 1.6 into $L^p$-bounds on various curvature tensors, and to consider our initial manifold to be a ball minus its center or slight generalizations (in other words, we assume that the topology of the underlying manifold is already known). In this article, we focus on $L^p$-bounds on the Bach tensor, namely we consider a metric *g* that is $C^{\infty }$ on $\mathbb {B}^4\setminus \{0\}$ with Riemann tensor in $L^2(\mathbb {B}^g,g)$ and Bach tensor in $L^p(\mathbb {B}^4,g)$ for some p>1. As opposed to Theorem 1.6, we do not assume any pointwise estimate on the metric near the singularity. Moreover, as though the extension of the metric across the singularity in Theorem 1.6 can be guessed using the asymptotic behaviour $g_{ij}(z)\underset{z\rightarrow \infty }{\sim } \delta _{ij}$, we do not have any a priori candidate of an extension that would satisfy better regularity properties in our setting. Therefore, we need to rely on the analysis of the partial differential equations induced by the assumption that the Bach tensor lies in $L^p$. We prove the following result.

### Theorem 1.7

Consider a $C^{\infty }$ orbifold metric *g* on $X{:}{=}C(\mathbb {S}^3/\Gamma )\setminus \{0\}$^2^, where Γ is a finite subgroup of $\textrm{SO}\left( 4,\mathbb {R} \right)$ and $C(\mathbb {S}^3/\Gamma )$ is the cone over $\mathbb {S}^3/\Gamma$. Assume that *g* satisfies the following assumptions:

1. The volume of *X* is finite: $\textrm{vol}_g(X)<+\infty$.
2. We have $\textrm{Riem}^g\in L^2(X,g)$ and $B^g\in L^p(X,g)$.
3. There exists a constant $\gamma _S>0$ such that the following Sobolev inequality holds:
  $$\begin{aligned} \forall u\in C^{\infty }_c(X),\qquad \Vert u\Vert _{L^4(X,g)}^2 \le \gamma _S \int _{X} |\nabla u|^2_g \, d\textrm{vol}_g. \end{aligned}$$
4. There exists a constant $\gamma _L>0$ such that the following regularity estimate holds. For any $u\in C^{\infty }_c(X\setminus \{0\})$, it holds
  $$\begin{aligned} \Vert \nabla u\Vert _{L^4(X,g)} \le \gamma _L \Vert \Delta _g u\Vert _{L^2(X,g)} . \end{aligned}$$

There exists a constant ε>0 depending only on $\textrm{vol}_g(X),\gamma _S,\gamma _L$ such that the following holds. Assume that

$$\begin{aligned} \Vert \textrm{Riem}^g\Vert _{L^2(X,g)} \le \varepsilon . \end{aligned}$$

Then there exist a diffeomorphism $\psi :X\rightarrow X$, a conformal factor $u\in C^{\infty }(X)$, and a $W^{2,2p}$ orbifold metric *h* on $C(\mathbb {S}^3/\Gamma )$ which is smooth on *X* such that

$$\begin{aligned} \psi ^*g = e^{2u}h. \end{aligned}$$

To the best of the authors’ knowledge, this is the first time that a background metric is constructed in a generic manner in the context of Huber’s theorem in dimension higher than 2. We can separate the assumptions of Theorem 1.7 into two categories: on the one hand (1) and (2) are natural geometric assumptions, on the other hand (3) and (4) are analytical assumptions which permit to transform the geometric assumptions into estimates. These analytical assumptions are satisfied on any "reasonable" manifold, for instance, after compactification of an asymptotically flat manifold. We discuss them in Section 2.1. Moreover, they are all satisfied by a $W^{2,2p}$-metric, which implies that our assumptions are minimal in some sense. As a consequence of Theorem 1.7, we prove the following version of Herzlich’s result. The second part can also be considered as an improvement of ε-regularity of Bach-flat metric of Tian and Viaclovsky, see Theorem 1.1 in [59].

### Corollary 1.8

Let $(M^4,g)$ be an asymptotically flat Riemannian manifold with the Riemann tensor in $L^2(M)$ and the Bach tensor in $L^p(M)$ for some p>1. Then (*M*, *g*) is obtained via stereographic projection from a compact $W^{2,2p}$-class Riemannian manifold (and in particular the $C^{0,\alpha }$-class for some α>0 depending on *p*). In particular, if (*M*, *g*) is Bach-flat, it can be obtain via stereographic projection from a compact $W^{2,2p}$-class Riemannian manifold for any p>1, and therefore also $C^{0,\alpha }$ for any 0<α<1.

Theorem 1.7 also applies to the extrinsic setting. Indeed, by Gauss–Codazzi equations, we know that the Riemann tensor is quadratic in the second fundamental form of the given immersion. Thus, the Bach tensor depends on the second derivatives of the second fundamental form. We obtain the following result, which can be understood as a study of branch points of immersions having a second fundamental form in $W^{2,\frac{4}{3}+\varepsilon }$ for some ε>0.

### Theorem 1.9

Let $\varepsilon \in (0,\frac{1}{10})$ and d≥5 be an integer. There exists δ>0 depending only on *d* and ε such that the following holds. Consider $\Phi :\mathbb {B}^4\setminus \{0\}\rightarrow \mathbb {R}^d$ be a smooth immersion such that its second fundamental form $\mathrm {I\!I}$ satisfies

$$\begin{aligned} \int _{\mathbb {B}^4} |\nabla ^2\mathrm {I\!I}|^{\frac{4}{3}+\varepsilon }_{g_{\Phi }} + |\nabla \mathrm {I\!I}|^{2+\varepsilon }_{g_{\Phi }} + |\mathrm {I\!I}|^{4+\varepsilon }_{g_{\Phi }} +1\, d\textrm{vol}_{g_{\Phi }} <+\infty . \end{aligned}$$

Assume that

$$\begin{aligned} \int _{\mathbb {B}^4} |\mathrm {I\!I}|^4_{g_{\Phi }}\, d\textrm{vol}_{g_{\Phi }} <\delta . \end{aligned}$$

Then, there exist a diffeomorphism $f\in \textrm{Diff}(\mathbb {B}^4\setminus \{0\})$, a conformal factor $u\in C^{\infty }(\mathbb {B}^4\setminus \{0\})$ and a metric *h* on $\mathbb {B}^4$ of class $W^{2,2p}(\mathbb {B}^4)$ for some p=p(ε)>1 such that $g_{\Phi \circ f} = e^{2u} h$.

The main idea behind the proof of Theorem 1.7 is to consider the tractor bundle and find a Coulomb gauge as it can be done for Yang–Mills connections by minimizing the $L^2$-norm of the connections of this bundle. Indeed, Korzyński and Lewandowski [41] in 2003 (see also [33] for the formalism of tractor calculus) proved that Bach-flat metrics correspond to the conformal class whose tractor bundle is Yang–Mills. The heuristic is that if the curvature of a bundle is regular across the singularity, then one should be able to extend the bundle itself across the singularity. Starting from this, one can expect to find an equivalent formulation for the Coulomb gauge introduced by Uhlenbeck [62], see also the survey of Rivière [55].

In 2-dimensional problems, Coulomb frames have been introduced by Hélein to prove the regularity of harmonic maps [36]. He later proved in [37] that these frames could be used to construct conformal coordinates for surfaces with $L^2$ second fundamental form, see also the recent survey [42]. In dimension 4, one could also try to find a preferred frame that would provide the conformal coordinates with respect to a preferred metric. However, finding the right energy on frames to consider is unclear since minimizing the connection forms does not seem to produce an integrable frame because of the Weyl obstruction. Instead, we consider the tractor bundle as explained previously, and find an equivalent formulation for the Coulomb gauge. The condition for a connection D=d+A over some bundle on the unit ball $\mathbb {B}^4$ to be a Coulomb gauge is the condition $d^*A=0$. This system turns out to be the Euler–Lagrange system of the functional $\int _{\mathbb {B}} |A|^2$. In the case of the tractor bundle, the connection is mainly given by the Schouten tensor. We refer to [25, 63] for an introduction to tractor calculus. Thanks to Chern–Gauss–Bonnet theorem in dimension 4, conformal variations of the $L^2$-norm of the Schouten tensor are equivalent to conformal variations of the $L^2$-norm of its trace. Hence, our problem of finding an optimal metric reduces to minimizing the $L^2$ norm of the scalar curvature in the conformal class of our given metric, see Section 2.3 for more details.

From a purely PDE view-point, the proof of Theorem 1.7 can be described as follows. One can understand the Bach tensor as the given data in a system satisfied by the Schouten tensor:

$$\begin{aligned} \Delta _g \textrm{Sch}^g = B^g + \textrm{Hess}_g J^g + \textrm{Riem}^g\star \textrm{Sch}^g, \end{aligned}$$

where $J^g= \textrm{tr}_g\, \textrm{Sch}^g$ and the term $\textrm{Riem}^g\star \textrm{Sch}^g$ stands for a linear combination of various contractions of the Riemann tensor $\textrm{Riem}^g$ and the Schouten tensor. Tracing this system provides no information since the Bach tensor is traceless (this is a consequence of the conformal invariance). Roughly, this means that the Bach tensor is actually the data in a system satisfied only by the traceless part of the Schouten tensor. In order to recover an information on the full tensor, one needs to prescribe the trace part $J^g$. In our setting, we work on degenerating annuli $\mathbb {B}\setminus \mathbb {B}_r$, with r→0. A first idea would be to solve a Yamabe problem (without caring too much about the boundary conditions) in order to find a metric $g_0 =e^{2u}g$ such that $J^{g_0}$ is constant. Then, the analysis would essentially be contained in the work of Tian and Viaclovsky [59, 60]. However, the resolution of such a problem requires some additional assumptions such as having a positive Yamabe invariant, see for instance [28, 49] and Remark 2.2. In some sense our ε-regularity, Theorem 2.13 comes as a generalization of the one of Tian–Viaclovsky, see Remark 2.14. In order to provide a general approach, one needs to solve an equation of higher order (an equation having the same number of derivative as $\Delta _g \textrm{Sch}^g$) and this is the reason of the assumptions (4) in Theorem 1.7. Our approach provides a metric $g_0=e^{2u} g$ which satisfies $\Delta _{g_0} J^{g_0} = 0$ and preserves a bound on the volume of $g_0$. Thus, $J^{g_0}$ is as regular as authorized by the geometric setting and the regularity of the metric $g_0$ is now fully determined by the Bach tensor $B^{g_0}=e^{-2u} B^g$. An interesting observation is that the *Q*-curvature of the metric $g_0$ now satisfies the pointwise bound $|Q^{g_0}|\le C|\textrm{Riem}^{g_0}|^2_{g_0}$ and thus, lies in $L^p(X,g_0)$ with p>1.

This strategy applies only to the dimension 4 since the Bach tensor is conformally invariant in dimension 4 and the Chern–Gauss–Bonnet formula is valid only in even dimension. However, a similar approach might be fruitful in higher dimension by considering the Fefferman–Graham obstruction tensors, see [29]. We also believe that this approach could be used in the same spirit as Coulomb gauges for Willmore surfaces [10, 45, 50], harmonic maps [44] and Yang–Mills connections [32, 55, 62], in order to study the regularity and compactness properties of Bach-flat metrics. Indeed, we are able to provide (at least locally) a conformal metric satisfying an ε-regularity property. It would be interesting to know whether we can solve $\Delta _{g_0} J^{g_0}=0$ globally.

It seems not possible to reduce the assumption $B^g\in L^p$ for p>1 to p=1. It would be interesting to look for possible counter-examples. Many directions of generalizations of Theorem 1.7 can be considered. First, one can study the higher regularity, as in [38], were we assume the Bach tensor to be in $W^{k,p}$ up to the singularity in some coordinates, and deduce that the background metric belongs to $W^{k+2,2p}$. Theorem 1.7 can be applied to Bach-flat metrics, but also the potential singularities arising along the Bach flow [8, 23] since it is valid for a Bach tensor in $L^2$. Another important direction, would be to integrate by parts the Bach tensor in order to obtain conditions on the Cotton tensor (which would ask for one derivative less). In particular, the same conclusion might still hold if instead of assuming $B^g\in L^p$, we assume $\textrm{Cot}^g\in L^{\frac{4p}{4-p}}$ for some p>1. This idea appeared recently in [7] concerning the Cotton tensor on 3-dimensional manifolds.

**Structure of the article:** In Section 2, we prove Theorem 1.7. In Section 3, we discuss the applications of Theorem 1.7 to asymptotically flat manifolds, and in particular the proof of Corollary 1.8. In Section 4, we prove Theorem 1.9.

## Huber Theorem for 4-Dimensional Manifolds

### Main Result

Consider *g* an orbifold metric on $C(\mathbb {S}^3/\Gamma )\setminus \{0\}$, where Γ is any finite subgroup of $\textrm{SO}\left( 4,\mathbb {R} \right)$. By definition, we can lift this metric to a smooth metric on $\mathbb {B}\setminus \{0\}$, where $\mathbb {B}$ is the unit ball of $\mathbb {R}^4$. Then, by working only in the space of functions which are invariant by Γ (finite group) which does not change the analysis, we can assume that $\Gamma =\{1\}$. Hence, the goal of this section is to prove the following result.

#### Theorem 2.1

Let p∈(1,2]. Consider a smooth metric *g* on $\mathbb {B}\setminus \{0\}$ satisfying the following assumptions:

1. The volume of $\mathbb {B}$ is finite: $\textrm{vol}_g(\mathbb {B})<+\infty$.
2. We have $\textrm{Riem}^g\in L^2(\mathbb {B},g)$ and $B^g\in L^p(\mathbb {B},g)$.
3. There exists a constant $\gamma _S>0$ such that the following Sobolev inequality holds:
  $$\begin{aligned} \forall u\in C^{\infty }_c(\mathbb {B}\setminus \{0\}),\quad \Vert u\Vert _{L^4(\mathbb {B})}^2 \le \gamma _S \int _{\mathbb {B}} |\nabla u|^2_g \, d\textrm{vol}_g. \end{aligned}$$
4. There exists a constant $\gamma _L>0$ such that the following regularity estimate holds. For any $u\in C^{\infty }_c(\mathbb {B}\setminus \{0\})$, it holds
  $$\begin{aligned} \Vert \nabla u\Vert _{L^4(\mathbb {B},g)} \le \gamma _L \Vert \Delta _g u\Vert _{L^2(\mathbb {B},g)} . \end{aligned}$$

There exists a constant ε>0 depending only on $\gamma _S$ and $\gamma _L$ such that the following holds. Assume that

$$\begin{aligned} \Vert \textrm{Riem}^g\Vert _{L^2(\mathbb {B},g)} \le \varepsilon . \end{aligned}$$

Then there exist a diffeomorphism $\psi :\mathbb {B}\setminus \{0\}\rightarrow \mathbb {B}\setminus \{0\}$, a conformal factor $u\in C^{\infty }(\mathbb {B}\setminus \{0\})$, and a metric $h\in W^{2,2p}(\mathbb {B},\xi )\cap C^{\infty }(\mathbb {B}\setminus \{0\})$ such that

$$\begin{aligned} \psi ^*g = e^{2u}h. \end{aligned}$$

Before proving the above result, we discuss all the assumptions of analytic nature. We will need to use Sobolev inequalities and elliptic regularity estimates for the metric *g*. To obtain uniform estimates, we need to assume that the constants are valid up to the origin.

**The isoperimetric inequality implies all the Sobolev embeddings.** The isoperimetric inequality is linked to Sobolev embeddings, see for instance [15]. From [21, Chapter IV, Section 3, Theorem 4], we know that

$$\begin{aligned} \Lambda ^4 {:}{=}\inf _{f\in C^{\infty }_c(\mathbb {B}\setminus \{0\})\setminus \{0\}} \frac{ \Vert \nabla f\Vert _{L^1(\mathbb {B},g)}^4 }{ \Vert f\Vert _{L^{\frac{4}{3}}(\mathbb {B},g)}^{3} } = \inf _{\Omega \subset \mathbb {B}\setminus \{0\}} \frac{\textrm{vol}(\partial \Omega )^4}{\textrm{vol}(\Omega )^3}. \end{aligned}$$

Hence, an isoperimetric inequality involves the following Sobolev inequality: for any $f\in C^{\infty }_c(\mathbb {B}\setminus \{0\})\setminus \{0\}$, it holds

1 $$\begin{aligned} \Vert f\Vert _{L^{\frac{4}{3}}(\mathbb {B},g)} \le \Lambda \, \Vert \nabla f\Vert _{L^1(\mathbb {B},g)}. \end{aligned}$$

By choosing $f= u^3$ for some $u\in C^{\infty }_c(\mathbb {B}\setminus \{0\})$, we obtain

$$\begin{aligned} \left( \int _{\mathbb {B}} u^4\, d\textrm{vol}_g \right) ^{\frac{3}{4}}&= \Vert u^3\Vert _{L^{\frac{4}{3}}(\mathbb {B},g)} \le 3\Lambda \int _{\mathbb {B}} |u|^2 |\nabla u|\, d\textrm{vol}_g\\&\le 3\Lambda \, \Vert u\Vert _{L^4(\mathbb {B},g)}^2\, \Vert \nabla u\Vert _{L^2(\mathbb {B},g)}. \end{aligned}$$

We deduce the following Sobolev inequality:

$$\begin{aligned} \Vert u\Vert _{L^4(\mathbb {B},g)} \le 3\, \Lambda \, \Vert \nabla u\Vert _{L^2(\mathbb {B},g)}. \end{aligned}$$

We now make another choice in (1). Namely,we chose $f=e^{\gamma |u|} -1$ for some γ>0 small to be chosen later, and $u\in C^{\infty }_c(\mathbb {B}\setminus \{0\})$ such that $\Vert \nabla u\Vert _{L^4(\mathbb {B},g)} = 1$, then it holds

$$\begin{aligned} \left\| e^{\frac{4\gamma }{3}|u|} \right\| _{L^1(\mathbb {B},g)}^{\frac{3}{4}}&\le \left\| e^{\gamma |u|} - 1\right\| _{L^{\frac{4}{3}}(\mathbb {B},g)} + \textrm{vol}_g(\mathbb {B})^{\frac{3}{4}} \\&\le \Lambda \gamma \int _{\mathbb {B}} e^{\gamma |u|} |\nabla u|\, d\textrm{vol}_g + \textrm{vol}_g(\mathbb {B})^{\frac{3}{4}}. \end{aligned}$$

By Hölder inequality and using the assumption $\Vert \nabla u\Vert _{L^4(\mathbb {B},g)} = 1$, we obtain

$$\begin{aligned} \left\| e^{\frac{4\gamma }{3}|u|} \right\| _{L^1(\mathbb {B},g)}^{\frac{3}{4}}\le \Lambda \gamma \left\| e^{\frac{4\gamma }{3}|u|} \right\| _{L^1(\mathbb {B},g)}^{\frac{3}{4}} + \textrm{vol}_g(\mathbb {B})^{\frac{3}{4}}. \end{aligned}$$

Chosing now $\gamma = (2\Lambda )^{-1}$, we obtain

$$\begin{aligned} \left\| e^{\frac{4\gamma }{3}|u|} \right\| _{L^1(\mathbb {B},g)}^{\frac{3}{4}}\le 2\textrm{vol}_g(\mathbb {B})^{\frac{3}{4}}. \end{aligned}$$

**Sobolev inequalities for hypersurfaces.** For the applications to generalized Willmore hypersurfaces, we can rely on Sobolev inequalities on hypersurfaces of $\mathbb {R}^5$, see for instance [14, Theorem 1.1], [3, Section 7], [51, Theorem 2.1] or [13, Theorem 1]. For any hypersurface $\Sigma ^4\subset \mathbb {R}^d$ and p∈[1,4), there exists a constant C=C(p,d)>0 such that

$$\begin{aligned} \forall \varphi \in C^\infty _c(\Sigma ),\qquad \left( \int _{\Sigma } |\varphi |^{\frac{4p}{4-p}}\, d\textrm{vol}_{\Sigma } \right) ^{\frac{4-p}{4}} \le C(p)\int _\Sigma |\nabla ^{\Sigma } \varphi |^p + |H\varphi |^p\ d\textrm{vol}_\Sigma . \end{aligned}$$

By Hölder inequality and assuming that $\Vert H\Vert _{L^4(\Omega )}$ is small enough for some open set $\Omega \subset \Sigma$, we obtain the following Sobolev inequality:

2 $$\begin{aligned} \forall \varphi \in C^\infty _c(\Omega ),\qquad \Vert \varphi \Vert _{L^{\frac{4p}{4-p}}(\Omega )} \le 2\, C(p)\, \Vert \nabla ^\Sigma \varphi \Vert _{L^p(\Omega )}. \end{aligned}$$

**Elliptic regularity** The assumption A.4 can be understood as a consequence of the following estimate on the Green kernel *G* of the Laplacian $\Delta _g$ with Dirichlet boundary data. We define $G:(\mathbb {B}\setminus \{0\})^2 \rightarrow \mathbb {R}$ as the solution to the following equation: for any $y\in \mathbb {B}\setminus \{0\}$, the function $G_y{:}{=}G(\cdot ,y)$ satisfies

$$\begin{aligned} {\left\{ \begin{array}{ll} -\Delta _g G_y = 0 &  \text {in }\mathbb {B}\setminus \{0\},\\ G_y = 0 &  \text {on }\partial (\mathbb {B}\setminus \{0\}). \end{array}\right. } \end{aligned}$$

Then A.4 is a consequence of the existence of a constant C>0 such that

$$\begin{aligned} \forall y\in \mathbb {B}\setminus \{0\},\qquad \sup _{\lambda>0}\, \lambda ^{\frac{4}{3}}\, \textrm{vol}_g\Big (\left\{ x\in \mathbb {B}\setminus \{0\} : |d G_y(x)|_{g(x)} > \lambda \right\} \Big ) \le C. \end{aligned}$$

It is proved in [15] that such an estimate is a consequence of a Sobolev inequality. Similarly as for (2), we can also see the assumption A.4 as a localized version for a coercivity condition on the Paneitz operator

$$\begin{aligned} \forall f\in C^{\infty }(\mathbb {B}\setminus \{0\}), \qquad P_g(f) {:}{=}\Delta _g^2 f + \textrm{div}_g\left( 2\, \textrm{Ric}(\nabla ^g f,\cdot ) - \frac{2}{3}\, \textrm{Scal}^g\, \nabla ^g f \right) . \end{aligned}$$

Indeed, A.4 is a consequence of the following inequality together with a smallness assumption on $\Vert \textrm{Ric}\Vert _{L^2(\mathbb {B},g)}$

$$\begin{aligned} \forall f\in C^{\infty }_c(\mathbb {B}\setminus \{0\}),\qquad \int _{\mathbb {B}} f\, (P_g f)\ d\textrm{vol}_g \ge C\, \left( \int _{\mathbb {B}} |\nabla f|^4_g\, d\textrm{vol}_g \right) ^{\frac{1}{2}}. \end{aligned}$$

### Definition of the Schouten Tensor and Relations

In this section, we define the Schouten tensor and provide a few remarks on the Bach tensor.

Let *g* be a smooth metric on $\mathcal {M}= \mathbb {B}^4 \setminus \{0\}$. The Schouten tensor of *g* is given by

$$\begin{aligned} \textrm{Sch}_{ab}^g {:}{=}\frac{1}{2} \left( \textrm{Ric}^g_{ab} - \frac{\textrm{Scal}^g}{6} g_{ab}\right) ,  &   J^g {:}{=}\textrm{tr}_g\, \textrm{Sch}^g = \frac{1}{6}\, \textrm{Scal}^g. \end{aligned}$$

If $g_u {:}{=}e^{2u}g$ and $\Delta _g = (\nabla ^g)^a (\nabla ^g)_a$ is the Laplace–Beltrami operator, then it holds, see for instance [11, Theorem 1.159]:

3 $$\begin{aligned} \textrm{Sch}^{g_u}_{ab}&= \textrm{Sch}^g_{ab} - \nabla _a^g \nabla ^g_b u + (\nabla _a^g u)(\nabla _b^g u) - \frac{|du|^2_g}{2} g_{ab},\end{aligned}$$

4 $$\begin{aligned} J^{g_u}&= e^{-2u} \Big ( J^g - \Delta _g u - |du|^2_g \Big ). \end{aligned}$$

Using the Bach tensor, we now deduce the equation relating $\Delta \textrm{Sch}$ and $\textrm{Hess}(J)$ obtained in [59, Section 2.1]. This is the fundamental system used to obtain ε-regularity. For any metric *g*, it holds:

5 $$\begin{aligned} (\Delta _g \textrm{Sch}^g )_{ij}&= B^g_{ij} + \nabla _{ij}^g J^g + 4\, (\textrm{Sch}^g)_i^{\ p}\, \textrm{Sch}^g_{pj} - |\textrm{Sch}^g|^2_g\, g_{ij} - 2\, (\textrm{Sch}^g)^{pk}\, W^g_{kipj}, \end{aligned}$$

where $B^g$ is the Bach tensor. Thanks to the Chern–Gauss–Bonnet formula, the Euler–Lagrange equation of $\int |\textrm{Sch}|^2$ is the same as the Euler–Lagrange equation of $\int J^2$ when we restrict ourselves to a conformal class:

6 $$\begin{aligned} \begin{aligned}&32\pi ^2 \chi (\mathbb {B}\setminus \mathbb {B}_r) = \int _{\mathbb {B}\setminus \mathbb {B}_r} |W^g|^2_g + 8((J^g)^2 - |\textrm{Sch}^g|^2_g)\ d\textrm{vol}_g \\&+ 8\int _{\partial (\mathbb {B}\setminus \mathbb {B}_r)} \left( \frac{1}{2} \textrm{Scal}^g H - \textrm{Ric}^g(\nu ,\nu ) H - \textrm{Riem}^{\gamma }_{\ \alpha \beta \gamma } \mathrm {I\!I}^{\alpha \beta } + \frac{1}{3} H^3 -H|\mathrm {I\!I}|^2_g + \frac{2}{3} \textrm{tr}_{g_{|\partial \mathbb {B}_r}} (\mathrm {I\!I}^3) \right) \, d\textrm{vol}_g. \end{aligned} \end{aligned}$$

### Strategy

Consider a metric *g* on $\mathbb {B}\setminus \{0\}$. By (5), we have the following system where $\textrm{Riem}\star \textrm{Sch}$ stands for linear combinations of different contractions between the Riemann tensor and the Schouten tensor

7 $$\begin{aligned} \Delta _g \textrm{Sch}^g - \textrm{Hess}_g(J^g) = B^g + \textrm{Riem}\star \textrm{Sch}^g. \end{aligned}$$

Since the Bach tensor is traceless (due to the conformal invariance), this system can be roughly understood as providing informations only on the traceless part of the Schouten tensor. Hence, some information is missing by look at the Bach tensor only. As observed in [41, Equation (43)] and [33, Lemma 4.2], the tractor bundle of $(\mathbb {B}\setminus \{0\},g)$ is Yang–Mills if and only if $B^g = 0$. Furthermore, it was proved in [58, Theorem 1.1] or [55, Theorem VI.9], that point singularities are removable for Yang–Mills connection. Hence, one could hope to get a similar result for Bach-flat manifolds. To do so, we first need to identify the Coulomb condition. For Yang–Mills connections, this condition can be understood as finding a critical point of the $L^2$-norm of the connection. Thanks to [33, Equation (15)] and the Chern–Gauss–Bonnet formula (6), we are reduced to study the following energy:

$$\begin{aligned} \forall u \in W^{2,2}(\mathbb {B},g),\qquad E(u) = \frac{1}{2} \int _{\mathbb {B}} (J^{g_u})^2\, d\textrm{vol}_{g_u}, \end{aligned}$$

where $g_u = e^{2u}g$. We prove in Lemma 2.5 that the Euler–Lagrange equation of *E* is $\Delta _{g_u} J^{g_u} = 1$. In particular, we obtained that $g_u$ is smooth as soon as *g* is smooth in Lemma 2.6.

#### Remark 2.2

- The main term $\int (J^{g_u})^2$ is positive and minimized when $J^{g_u} = 0$. Marques [49, Theorem 1.2] proved that we can solve $\bar{J}=0$ under the condition that the first eigenvalue of the conformal Laplacian has to be positive, see for instance [28]. Without this condition, it is unclear that we can solve $J^{g_u} = 0$ with estimates on *u*.
- If $\bar{J}=0$ and $\bar{B}=0$, then the ε-regularity has been proved in [16, 59]. We need to bound the constant in Sobolev inequality, see for instance [2, 27], or an estimate on the growth of balls, see for instance [60].

However, solving this problem directly on $\mathbb {B}$ is not obvious. Instead, we first solve it in $\mathbb {B}\setminus \mathbb {B}_r$, that is to say, we minimize the following functional:

$$\begin{aligned} \forall u \in W^{2,2}_0(\mathbb {B}\setminus \mathbb {B}_r,g),\qquad E_r(u) = \frac{1}{2}\int _{\mathbb {B}\setminus \mathbb {B}_r} (J^{g_u})^2\, d\textrm{vol}_{g_u}. \end{aligned}$$

Then we pass to the limit r→0. To do so, we need some preliminary estimates. We prove an ε-regularity in the spirit of [59, 60]. A priori, we have a system of the form Δu=fu+g. If *f* and *g* are regular, then *u* is regular as well. We then show that the limiting metric $g_0$ is continuous across the singularity by proceeding to a blow-up at the origin, see [5, 6]. In a neighbourhood of the origin, $(\mathbb {B}\setminus \{0\},\bar{g})$ will be diffeomorphic to a cone $C(\mathbb {S}^3/\Gamma )\setminus \{0\}$ where Γ is a finite subgroup of $\textrm{SO}\left( 4,\mathbb {R} \right)$. Since $\mathbb {B}$ is simply connected, we obtain that Γ is trivial. We can extend continuously $g_0$ to the origin by the flat metric, see [9, 57].

### The Energy Functional

In this section, we consider a smooth metric *g* on $\mathbb {B}\setminus \{0\}$ satisfying the assumptions of Theorem 2.1. We denote $\Omega _r {:}{=}\mathbb {B}^4\setminus \mathbb {B}_r^4$. If $u\in C^\infty (\Omega _r)$, we define $g_u {:}{=}e^{2u}g$. Given r>0, we define

8 $$\begin{aligned} \forall u\in C^\infty (\Omega _r),\qquad E_r(u) {:}{=}\frac{1}{2} \int _{\Omega _r } (J^u)^2\, d\textrm{vol}_{g_u}. \end{aligned}$$

#### Lemma 2.3

Up to replace $\mathbb {B}$ with $\mathbb {B}_{r_0}$ for some $r_0>0$ small enough, one can assume that

9 $$\begin{aligned} E_0(0) = \frac{1}{2}\int _{\mathbb {B}}(J^g)^2 \, d\textrm{vol}_g < \frac{1}{4^6\, \gamma _L^4}. \end{aligned}$$

Consider $(u_k)_{k\in \mathbb {N}} \subset C^\infty _c(\Omega _r)$ a minimizing sequence for the following optimization problem:

$$\begin{aligned}&\inf \left\{ E_r(w) : w\in C^\infty _c(\Omega _r),\ \Vert dw\Vert _{L^4(\Omega _r)}< \frac{1}{4\, \gamma _L} \right\} \\&\quad = \inf \left\{ E_r(w) : w\in W^{2,2}_0(\Omega _r,g),\ \Vert dw\Vert _{L^4(\Omega _r)} < \frac{1}{4\, \gamma _L} \right\} . \end{aligned}$$

Then $(u_k)_{k\in \mathbb {N}}$ weakly converges in $W^{2,2}_0$, up to a subsequence, to some $u_r\in W^{2,2}_0(\Omega _r,g)$ such that

$$\begin{aligned} E_r(u_r) = \inf \left\{ E_r(w) : w\in C^\infty _c(\Omega _r),\ \Vert dw\Vert _{L^4(\Omega _r)} < \frac{1}{4\, \gamma _L}\right\} . \end{aligned}$$

In particular, $u_r$ is a critical point of $E_r$ in $W^{2,2}_0(\Omega _r,g)$ satisfying the following estimates:

10 $$\begin{aligned} \left\{ \begin{aligned}&\Vert du_r\Vert _{L^4(\Omega _r,g)} < \frac{8}{3}\, \gamma _L\, E_r(0)^{\frac{1}{2}},\\&\Vert \Delta _g u_r\Vert _{L^2(\Omega _r,g)} \le \Vert du_r\Vert _{L^4(\Omega _r,g)}^2 + 2\, E_r(0)^{\frac{1}{2}}. \end{aligned} \right. \end{aligned}$$

#### Proof

From(4), it holds

$$\begin{aligned} \forall k\in \mathbb {N},\qquad E_r(u_k) = \frac{1}{2}\int _{\Omega _r} \left( J^g - \Delta _g u_k - |du_k|^2_g \right) ^2 \, d\textrm{vol}_g\le E_r(0). \end{aligned}$$

Since the exponential term is positive, we obtain

$$\begin{aligned} \left\| J^g - \Delta _g u_k - |du_k|^2_g \right\| _{L^2(\Omega _r,g)} \le \left( 2\, E_r(0)\right) ^{\frac{1}{2}}. \end{aligned}$$

Using the triangle inequality, we deduce

$$\begin{aligned} \Vert \Delta _g u_k\Vert _{L^2(\Omega _r,g)}&\le \Vert J^g\Vert _{L^2(\Omega _r,g)} + \Vert du_k\Vert _{L^4(\Omega _r,g)}^2 +\left( 2\, E_r(0)\right) ^{\frac{1}{2}} \\&\le \Vert du_k\Vert _{L^4(\Omega _r,g)}^2 + 2^{\frac{3}{2}} E_r(0)^{\frac{1}{2}}. \end{aligned}$$

Since $\Vert du_k\Vert _{L^4(\Omega _r,g)} < (4\gamma _L)^{-1}$, we obtain

11 $$\begin{aligned} \Vert \Delta _g u_k\Vert _{L^2(\Omega _r,g)}&\le \frac{1}{4\, \gamma _L}\Vert du_k\Vert _{L^4(\Omega _r,g)} + 4 E_r(0)^{\frac{1}{2}}. \end{aligned}$$

Thanks to A.4, we obtain

$$\begin{aligned} \Vert du_k\Vert _{L^4(\Omega _r,g)}&\le \frac{1}{4}\Vert du_k\Vert _{L^4(\Omega _r,g)} + 4\, \gamma _L\, E_r(0)^{\frac{1}{2}}. \end{aligned}$$

Hence, it holds

$$\begin{aligned} \Vert du_k\Vert _{L^4(\Omega _r,g)} \le \frac{16}{3} \gamma _L\, E_r(0)^{\frac{1}{2}}. \end{aligned}$$

Thanks to (9), we obtain:

$$\begin{aligned} \Vert du_k\Vert _{L^4(\Omega _r,g)} \le \frac{1}{12\, \gamma _L}. \end{aligned}$$

Together with (11), we deduce that $(u_k)_{k\in \mathbb {N}}$ remains bounded in $W^{2,2}_0(\Omega _r,g)$ and also in the interior of the ball $\mathcal {B}{:}{=}\left\{ w\in W^{1,4}_0(\Omega _r): \Vert dw\Vert _{L^4(\Omega _r,g)} < (4 \gamma _L)^{-1} \right\}$. □

#### Remark 2.4

Since $u_r=0$ on $\partial \Omega _r$, $u_r$ also minimize the $L^2$-norm of $\textrm{Sch}$ thanks to the Chern–Gauss–Bonnet formula (6). Thus, if $g_r {:}{=}g_{u_r}$ then we have the following uniform bound:

$$\begin{aligned} \int _{\Omega _r} |\textrm{Riem}^{g_r}|^2_{g_r}\, d\textrm{vol}_{g_r}&\le \int _{\mathbb {B}\setminus \mathbb {B}_r} |\textrm{Riem}^{g}|^2_{g}\, d\textrm{vol}_{g} \le \int _{\mathbb {B}\setminus \{0\}} |\textrm{Riem}^{g}|^2_{g}\, d\textrm{vol}_{g}. \end{aligned}$$

In the following lemma, we compute the Euler–Lagrange equation of $E_r$.

#### Lemma 2.5

Let $u\in W^{2,2}_0(\Omega _r,g)$ be a critical point of $E_r$. Then, the metric $g_u$ satisfies the following equation:

$$\begin{aligned} \Delta _{g_u} J^{g_u} = 0 \qquad \text {in }\mathcal {D}'(\Omega _r). \end{aligned}$$

#### Proof

Consider a variation $(v_t)_{t\in (-1,1)}$ of *u* with $v_0 = u$ and $\dot{v} {:}{=}(\partial _t v_t)|_{t=0}$ having compact support in $\Omega _r$. We compute the first derivative thanks by using $g_{v_t} = e^{2v_t} g$ and the formula (4):

12 $$\begin{aligned} 0 = \left. \frac{d E_r(v_t)}{dt}\right| _{t=0}&= \frac{d}{dt}\left. \left( \frac{1}{2}\int _{\Omega _r} \left( J^g - \Delta _g v_t - |dv_t|^2_g \right) ^2 \, d\textrm{vol}_g \right) \right| _{t=0} \nonumber \\&= \int _{\Omega _r} \left( J^g - \Delta _g u - |du|^2_g \right) \left( -\Delta _g \dot{v} - 2\left\langle du,d\dot{v} \right\rangle _g \right) \, d\textrm{vol}_g \nonumber \\&= \int _{\Omega _r} e^{2u} J^{g_u}\left( -\Delta _g \dot{v} - 2\left\langle du,d\dot{v} \right\rangle _g \right) \, d\textrm{vol}_g. \end{aligned}$$

After integration by parts, we obtain:

$$\begin{aligned} 0 = \int _{\Omega _r} \dot{v}\left( -\Delta _g(e^{2u}J^{g_u}) + 2\, \textrm{div}_g(e^{2u}J^{g_u} \nabla ^g u) \right) \, d\textrm{vol}_g. \end{aligned}$$

Since this is valid for every $\dot{v}\in W^{2,2}_0$, we obtain:

$$\begin{aligned} 0&= -\Delta _g(e^{2u}J^{g_u}) + 2\, \textrm{div}_g(e^{2u}J^{g_u} \nabla ^g u) \\&= -\textrm{div}_g\left( 2\, e^{2u}\, (\nabla ^g u)\, J^{g_u} + e^{2u}\, \nabla ^g J^{g_u} \right) + 2 \textrm{div}_g(e^{2u}\, J^{g_u}\, \nabla ^g u) \\&= -\textrm{div}_g \left( e^{2u}\, \nabla ^g J^{g_u} \right) \\&= -\textrm{div}_g\left( e^{4u}\, \nabla ^{g_u} J^{g_u} \right) \\&= -e^{4u} \Delta _{g_u} J^{g_u}. \end{aligned}$$

□

Since *g* is of class $C^{\infty }$, we obtain that $u_r$ is also of class $C^{\infty }$.

#### Lemma 2.6

Let $u\in W^{2,2}_0(\Omega _r,g)$ be a critial point of $E_r$ obtained in Lemma 2.3. It holds $u\in C^\infty (\Omega _r)\cap C^0\left( \overline{\Omega _r}\right)$. Moreover, for any $\frac{1}{2}>s>r>0$ and $k\in \mathbb {N}$, there exist C>1 depending only on *s*, *k*, $\gamma _L$, $\gamma _S$, $\max _{1\le i,j\le 4}\Vert g_{ij}\Vert _{C^{k+2}(\Omega _s)}$ and $\max _{1\le i,j\le 4}\Vert g^{ij}\Vert _{C^{k+2}(\Omega _s)}$ such that

13 $$\begin{aligned} \Vert u\Vert _{C^k(\Omega _{2s})} \le C. \end{aligned}$$

#### Remark 2.7

The constant *C* might blow up as s→0.

#### Proof

Thanks to (12), the Euler–Lagrange equation of $E_r$ can also be written as:

14 $$\begin{aligned} -\Delta _g\left( J^g - \Delta _g u - |du|^2_g \right) +2\, \textrm{div}_g\left( (J^g - \Delta _g u - |du|^2_g)\, \nabla ^g u \right) = 0. \end{aligned}$$

Hence the function $v {:}{=}J^g - \Delta _g u - |du|^2_g$ satisfies:

$$\begin{aligned} \forall p\in [1,\infty ),\qquad \Delta _g v \in W^{-1,(\frac{4}{3},1)}(\Omega _r,g). \end{aligned}$$

Indeed, it holds $du \in L^{(4,2)}(\Omega _r)$, so that $(\Delta _g u)\, du \in L^{(2,1)}(\Omega _r)$. We also have $du \in L^{(4,3)}(\Omega _r)$, so that $|du|^3 \in L^{(\frac{4}{3},1)}(\Omega _r)$. Hence, it holds $v \in W^{1,(\frac{4}{3},1)}(\Omega _r)\hookrightarrow L^{(2,1)}(\Omega _r)$, meaning that $\Delta _g u \in L^{(2,1)}(\Omega _r)$. Thus, we obtain $u\in W^{2,(2,1)}(\Omega _r)\hookrightarrow C^0\left( \overline{\Omega _r}\right)$. Consequently, the function $J^{g_u} = e^{2u}v\in W^{1,\frac{4}{3}}(\Omega _r)$ is a solution to

$$\begin{aligned} \partial _{\alpha } \left( e^{2u} g^{\alpha \beta }\, \sqrt{\det g}\, \partial _\alpha J^{g_u} \right) = 0. \end{aligned}$$

We now have $e^{2u} g^{\alpha \beta }\, \sqrt{\det g} \in W^{1,(4,1)}(\Omega _r) \subset C^0\left( \overline{\Omega _r}\right)$. By a straightforward adaptation of the proof of [4, Theorem A1.1], we deduce that $J^{g_u} \in W^{1,p}$ for every p>1. We consider now the following equation:

$$\begin{aligned} \Delta _g (e^u) = \left( e^{2u}J^{g_u} - J^g\right) e^u. \end{aligned}$$

The right-hand side belongs to $W^{1,4-\varepsilon }(\Omega _r)$ for every $\varepsilon \in (0,1)$. Thus, $e^u \in W^{2,4-\varepsilon }(\Omega _r)$ for every $\varepsilon \in (0,1)$. Thanks to Sobolev embeddings, we deduce that $e^u \in W^{1,p}(\Omega _r)$ for every $p\in [1,\infty )$. Since *u* is continuous, we deduce that $u\in W^{1,p}(\Omega _r)$ for every $p\in [1,\infty )$. We now come back to the equation:

$$\begin{aligned} \Delta _g u = |du|^2_g + e^{2u} J^{g_u} - J^g. \end{aligned}$$

We deduce that $u\in W^{3,p}(\Omega _r)$ for every $p\in [1,\infty )$. By a bootrstrap argument, we obtain $u\in C^\infty (\Omega _r)$. □

### Sobolev Inequalities and Integrability of the Curvature Tensors in the New Metric

Thanks to Lemma 2.6, the metrics $g_{u_r} = e^{2u_r}\, g$ obtained in Lemma 2.3 are uniformly bounded in $C^k_{\textrm{loc}}(\mathbb {B}\setminus \{0\})$ for any k≥1. Up to a subsequence, we can pass to the limit r→0 and we obtain metric $g_0 {:}{=}e^{2u}g$ which is $C^{\infty }$ on $\Omega _0 {:}{=}\mathbb {B}\setminus \{0\}$ and satisfies $\Delta _{g_0}J^{g_0}=0$ on $\Omega _0$. In this section, we prove some regularity estimates on the conformal factor *u* in Lemma 2.8. We deduce some integrability of $B^{g_0}$ in Lemma 2.9 and the Sobolev inequalities in Lemma 2.10.

Given $\varphi \in C^\infty _c(\mathbb {B}\setminus \{0\})$, we denote

$$\begin{aligned} (\varphi )_{\mathbb {B}} {:}{=}\frac{1}{\textrm{vol}_g(\mathbb {B}\setminus \{0\})} \int _{\mathbb {B}\setminus \{0\}} \varphi \, d\textrm{vol}_g. \end{aligned}$$

We first prove that $e^{-u}\in W^{1,2}(\mathbb {B},g)$.

#### Lemma 2.8

Let $\gamma _L>1$ and $\gamma _S>0$ be defined in A.4 and A.3. Assume that

15 $$\begin{aligned} \int _{\mathbb {B}} \frac{(J^g)^2}{2} \, d\textrm{vol}_g \le \frac{9}{4^5 \gamma ^2_S}. \end{aligned}$$

Then, it holds $e^{-u}\in W^{1,2}(\mathbb {B},g)$ with the following estimates:

$$\begin{aligned} {\left\{ \begin{array}{ll} \displaystyle \Vert e^{-u}\Vert _{L^4(\mathbb {B},g)}^2 \le \frac{1}{4 \gamma _L^2}+ 8\, \textrm{vol}_g(\mathbb {B})^{\frac{1}{2}},\\ \displaystyle \Vert d(e^{-u})\Vert _{L^2(\mathbb {B},g)}^2 \le \frac{1}{8 \gamma _S^2}\left( \frac{1}{2 \gamma _L^2} + 8\textrm{vol}_g(\mathbb {B})^{\frac{1}{2}} \right) . \end{array}\right. } \end{aligned}$$

#### Proof

Given r>0, we prove the following estimates for $u_r$ and then pass to the limit r→0: it holds $e^{-u_r}\in W^{1,2}(\Omega _r,g)$ with the following estimates

$$\begin{aligned} \Vert e^{-u_r}\Vert _{L^4(\Omega _r,g)}^2 \le \frac{\gamma _S}{24\gamma _L^4},  &   \Vert d(e^{-u_r})\Vert _{L^2(\Omega _r,g)}^2 \le \frac{1+\gamma _S}{32\gamma _L^4}. \end{aligned}$$

We compute the equation satisfied by $e^{-u_r}$:

$$\begin{aligned} \Delta _g\left( e^{-u_r} \right)&= -\textrm{div}_g\left( e^{-u_r} \nabla ^g u_r\right) \\&= e^{-u_r}|du_r|^2_g -e^{-u_r} (\Delta _g u_r) \\&= e^{-u_r} |du_r|^2_g + e^{-u_r}\left( |du_r|^2_g + e^{2u_r} J^{g_r} - J^g \right) \\&= 2e^{-u_r} |du_r|^2_g + e^{-u_r}\left( e^{2u_r} J^{g_r} - J^g \right) . \end{aligned}$$

We multiply by $e^{-u_r}$ and integrate by parts:

$$\begin{aligned}&\int _{\partial \Omega _r} e^{-2u_r} (\partial _\nu u_r) d\textrm{vol}_g - \int _{\Omega _r} |d(e^{-u_r})|^2_g\, d\textrm{vol}_g \\&\quad =\ 2\int _{\Omega _r} e^{-2u_r} |du_r|^2_g + e^{-2u_r} \left( e^{2u_r} J^{g_r} - J^g \right) \, d\textrm{vol}_g. \end{aligned}$$

We consider the first term of the left-hand side: since $u_r = 0$ on $\partial \Omega _r$, it holds

$$\begin{aligned} \int _{\partial \Omega _r} e^{-2u_r} (\partial _\nu u_r) d\textrm{vol}_g&= \int _{\partial \Omega _r} (\partial _\nu u_r) d\textrm{vol}_g \\&=\int _{\Omega _r} \Delta _g u_r\, d\textrm{vol}_g \\&= \int _{\Omega _r} -|du_r|^2_g - e^{2u_r}J^{g_r} + J^g\, d\textrm{vol}_g. \end{aligned}$$

We end up with the following estimate:

$$\begin{aligned}&3\int _{\Omega _r} |d(e^{-u_r})|^2\, d\textrm{vol}_g\\&\quad = - \int _{\Omega _r} e^{-2u_r} \left( e^{2u_r} J^{g_r} - J^g \right) \, d\textrm{vol}_g -\int _{\Omega _r} |du_r|^2_g + e^{2u_r}J^{g_r} - J^g\, d\textrm{vol}_g \\&\quad \le \ \Vert e^{-2u_r} \Vert _{L^2(\Omega _r,g)}\Vert e^{2u_r} J^{g_r} - J^g\Vert _{L^2(\Omega _r,g)}\\&\qquad + \textrm{vol}_g(\Omega _r)^{\frac{1}{2}} \left( \Vert du_r\Vert _{L^4(\Omega _r,g)}^2 + \Vert e^{2u_r} J^{g_r} - J^g\Vert _{L^2(\Omega _r,g)} \right) \\&\quad \le \ 4 E_r(0)^{\frac{1}{2}} \left( \Vert e^{-u_r}\Vert _{L^4(\Omega _r,g)}^2 + \Vert du_r\Vert _{L^4(\Omega _r,g)}^2 + E_r(0)^{\frac{1}{2}} \right) . \end{aligned}$$

From Lemma 2.3 and (9), we obtain:

16 $$\begin{aligned} 3\int _{\Omega _r} |d(e^{-u_r})|^2\, d\textrm{vol}_g \le 4 E_r(0)^{\frac{1}{2}} \left( \Vert e^{-u_r}\Vert _{L^4(\Omega _r,g)}^2 + \frac{1}{4 \gamma _L^2} \right) . \end{aligned}$$

Thanks to Item A.3, it holds

$$\begin{aligned} \Vert e^{-u_r}-1\Vert _{L^4(\Omega _r,g)}^2 \le \frac{4\gamma _S}{3} E_r(0)^{\frac{1}{2}} \left( \Vert e^{-u_r}\Vert _{L^4(\Omega _r,g)}^2 + \frac{1}{4 \gamma _L^2} \right) . \end{aligned}$$

Thanks to (15), we obtain

$$\begin{aligned} \Vert e^{-u_r}\Vert _{L^4(\Omega _r,g)}^2&\le 4\Vert e^{-u_r}-1\Vert _{L^4(\Omega _r,g)}^2 + 4\, \textrm{vol}_g(\Omega _r)^{\frac{1}{2}}\\&\le 4\left( \frac{4\gamma _S}{3} E_r(0)^{\frac{1}{2}} \Vert e^{-u_r}\Vert _{L^4(\Omega _r,g)}^2 + \frac{1}{32 \gamma _L^2} \right) + 4\, \textrm{vol}_g(\Omega _r)^{\frac{1}{2}} \\&\le \frac{1}{2}\Vert e^{-u_r}\Vert _{L^4(\Omega _r,g)}^2 + \frac{1}{8 \gamma _L^2}+ 4\, \textrm{vol}_g(\Omega _r)^{\frac{1}{2}}. \end{aligned}$$

Hence, it holds

$$\begin{aligned} \Vert e^{-u_r}\Vert _{L^4(\Omega _r,g)}^2 \le \frac{1}{4 \gamma _L^2}+ 8\, \textrm{vol}_g(\Omega _r)^{\frac{1}{2}}. \end{aligned}$$

Coming back to (16) and using $\gamma _L>1$, we obtain

$$\begin{aligned} \Vert d(e^{-u_r})\Vert _{L^2(\Omega _r,g)}^2 \le \frac{1}{8 \gamma _S^2}\left( \frac{1}{2 \gamma _L^2} + 8\, \textrm{vol}_g(\Omega _r)^{\frac{1}{2}} \right) . \end{aligned}$$

□

We now show that the Bach tensor $B^{g_0}$ satisfies certain integrability.

#### Lemma 2.9

For any $q\in [1,+\infty )$, we have the following estimate:

$$\begin{aligned} \left\| B^{g_0} \right\| _{L^{\frac{2q}{1+q}}(\mathbb {B},g_0)} \le \left( \frac{1}{4 \gamma _L^2}+ 8\, \textrm{vol}_g(\mathbb {B})^{\frac{1}{2}} \right) ^{\frac{q-1}{4q}} \left\| B^g \right\| _{L^q(\mathbb {B},g)}. \end{aligned}$$

#### Proof

The Bach tensor satisfies the following transformation formula:

$$\begin{aligned} B^{g_0}_{ij} = e^{-2u} B^g_{ij}. \end{aligned}$$

Thus, it holds $|B^{g_0}|_{g_0} = e^{-4u} |B^g|_g$. If q≥1, we have $\frac{q-1}{q+1} + \frac{2}{q+1}=1$. Thus, we obtain

$$\begin{aligned} \int _{\mathbb {B}} |B^{g_0}|^{\frac{2q}{1+q}}_{g_0}\, d\textrm{vol}_{g_0}&= \int _{\mathbb {B}} e^{4\left( 1-\frac{2q}{1+q} \right) u}\, |B^g|^{\frac{2q}{1+q}}_g\, d\textrm{vol}_g \\&= \int _{\mathbb {B}} e^{4\frac{1-q}{1+q} u_r}\, |B^g|^{\frac{2q}{1+q}}_g\, d\textrm{vol}_g \\&\le \left\| e^{-4\frac{q-1}{1+q}u} \right\| _{L^{\frac{q+1}{q-1}}(\mathbb {B},g)} \left\| |B^g|^{\frac{2q}{1+q}}_g \right\| _{L^{\frac{q+1}{2}}(\mathbb {B},g)} \\&\le \left\| e^{-u} \right\| _{L^4(\mathbb {B},g)}^{\frac{q-1}{q+1}} \left\| B^g \right\| _{L^q(\mathbb {B},g)}^{\frac{2q}{q+1}}. \end{aligned}$$

We conclude thanks to Lemma 2.8. □

We deduce from Lemma 2.8 a Sobolev inequality for the metric $g_r$.

#### Lemma 2.10

Assume that

17 $$\begin{aligned} \int _{\mathbb {B}} \frac{(J^g)^2}{2}\, d\textrm{vol}_g \le \left( \frac{3}{8\, \gamma _L} \right) ^2\, \frac{1}{4\, \gamma _S^2}. \end{aligned}$$

Then, for any $\psi \in C^\infty _c(\mathbb {B}\setminus \{0\})$, it holds

$$\begin{aligned} \left( \int _{\mathbb {B}} \psi ^4\, d\textrm{vol}_{g_0} \right) ^{\frac{1}{2}} \le 4\gamma _S \int _{\mathbb {B}} |d\psi |^2_{g_0}\, d\textrm{vol}_{g_0}. \end{aligned}$$

#### Proof

Thanks to (10), we have

18 $$\begin{aligned} \int _{\mathbb {B}} |du|^4_g\, d\textrm{vol}_g \le \frac{1}{4\gamma ^2_S}. \end{aligned}$$

Let $\psi \in C^\infty _c(\mathbb {B}\setminus \{0\})$ and $\varphi {:}{=}e^{u}\psi$. From A.3, it holds

$$\begin{aligned} \left( \int _{\mathbb {B}} e^{4u} \psi ^4\, d\textrm{vol}_g\right) ^{\frac{1}{2}} \le \gamma _S \int _{\mathbb {B}} |d(e^{u}\psi )|^2_g \, d\textrm{vol}_g. \end{aligned}$$

We write the above inequality in terms of the metric $g_r$:

$$\begin{aligned} \left( \int _{\mathbb {B}} \psi ^4\, d\textrm{vol}_{g_0} \right) ^{\frac{1}{2}}&\le \gamma _S \int _{\mathbb {B}} 2\psi ^2 e^{2u}|du|^2_g +2e^{2u} |d\psi |^2_g\, d\textrm{vol}_g\\&\le 2\gamma _S\int _{\mathbb {B}} |d\psi |^2_{g_0}\, d\textrm{vol}_{g_r} + 2\gamma _S \int _{\Omega _r} \psi ^2 e^{2u}|du|^2_g\, d\textrm{vol}_g \\&\le 2\gamma _S\int _{\Omega _r} |d\psi |^2_{g_0}\, d\textrm{vol}_{g_0} + 2\gamma _S \left( \int _{\mathbb {B}} \psi ^4 \, d\textrm{vol}_{g_0} \right) ^{\frac{1}{2}} \Vert du\Vert _{L^4(\mathbb {B},g)}^2. \end{aligned}$$

Thanks to (18), we obtain

$$\begin{aligned} \left( \int _{\mathbb {B}} \psi ^4\, d\textrm{vol}_{g_0} \right) ^{\frac{1}{2}} \le 2\gamma _S\int _{\mathbb {B}} |d\psi |^2_{g_0}\, d\textrm{vol}_{g_0} + \frac{1}{2} \left( \int _{\mathbb {B}} \psi ^4\, d\textrm{vol}_{g_0} \right) ^{\frac{1}{2}}. \end{aligned}$$

We obtain the desired result by reabsorbing the last term of the left-hand side with the right-hand side. □

We now prove a volume bound for the metric $g_0$.

#### Lemma 2.11

Under the assumption (17), it holds

$$\begin{aligned} \textrm{vol}_{g_0}(\mathbb {B}) \le 2\, (2\, \gamma _S)^{\frac{1}{2}}\, \textrm{vol}_g(\mathbb {B})^{\frac{1}{4}}. \end{aligned}$$

#### Proof

It suffices to prove that $e^u\in L^4(\mathbb {B},g)$. To do so, we use the construction $u=\displaystyle \lim _{r\rightarrow 0}u_r$ and the fact that $u_r=0$ on $\partial (\mathbb {B}\setminus \mathbb {B}_r)$. We denote $\Omega _r{:}{=}\mathbb {B}\setminus \mathbb {B}_r$. Let δ>0 a parameter to be chosen later and use the Sobolev inequality A.3 to the function

$$\begin{aligned} v{:}{=}\exp \left( \delta \frac{|u_r|}{\Vert \nabla u_r\Vert _{L^4(\mathbb {B},g)}}\right) \in 1+ W^{1,2}_0(\Omega _r,g). \end{aligned}$$

It holds

$$\begin{aligned} \left\| v \right\| _{L^4(\mathbb {B}\setminus \mathbb {B}_r,g)}&\le \left\| v - 1 \right\| _{L^4(\mathbb {B}\setminus \mathbb {B}_r,g)} + \textrm{vol}_g(\mathbb {B})^{\frac{1}{4}} \\&\le \sqrt{\gamma _S}\left\| \nabla v \right\| _{L^2(\mathbb {B}\setminus \mathbb {B}_r,g)}+ \sqrt{\gamma _S}\, \textrm{vol}_g(\mathbb {B})^{\frac{1}{4}}. \end{aligned}$$

By Hölder inequality, we obtain

$$\begin{aligned} \left\| v \right\| _{L^4(\mathbb {B}\setminus \mathbb {B}_r,g)}\le \delta \sqrt{\gamma _S}\left\| v \right\| _{L^4(\mathbb {B}\setminus \mathbb {B}_r,g)}+ \sqrt{\gamma _S}\, \textrm{vol}_g(\mathbb {B})^{\frac{1}{4}}. \end{aligned}$$

We choose $\delta = (2\gamma _S)^{-1/2}$ and we obtain

$$\begin{aligned} \left\| \exp \left( \frac{|u_r|}{(2\gamma _S)^{1/2}\Vert \nabla u_r\Vert _{L^4(\mathbb {B},g)}}\right) \right\| _{L^4(\mathbb {B}\setminus \mathbb {B}_r,g)}\le \frac{ \sqrt{2\, \gamma _S} }{ \sqrt{2}-1}\, \textrm{vol}_g(\mathbb {B})^{\frac{1}{4}}. \end{aligned}$$

By (17) and (10), we obtain $(2\gamma _S)^{1/2}\Vert \nabla u_r\Vert _{L^4(\mathbb {B}\setminus \mathbb {B}_r,g)} \le 1$, we obtain

$$\begin{aligned} \left\| e^{u_r} \right\| _{L^4(\mathbb {B}\setminus \mathbb {B}_r,g)} \le \left\| e^{|u_r|} \right\| _{L^4(\mathbb {B}\setminus \mathbb {B}_r,g)} \le 2\sqrt{2\, \gamma _S}\, \textrm{vol}_g(\mathbb {B})^{\frac{1}{4}}. \end{aligned}$$

□

### Regularity Estimates

In this section, we prove an ε-regularity estimate in Theorem 2.13, inspired by Moser’s iteration, see for instance [35, Theorem 4.4]. First, we need to prove some estimate on the derivatives of $J^{g_0}$.

#### Lemma 2.12

Let *g* a metric on $\mathbb {B}\setminus \{0\}$ satisfying the hypothesis of theorem 2.1, then there exists ε>0 and C>0 depending on the operation ⋆ in (7), *p* and $\gamma _S$ such that the following holds. Assume that

19 $$\begin{aligned} \Vert \textrm{Riem}^g\Vert _{L^2(\mathbb {B},g)} \le \varepsilon . \end{aligned}$$

Let $g_0$ be the metric conformal to *g* on $\Omega _0$ defined at the beginning of section 2.5 satisfying

$$\begin{aligned} \Delta _{g_0} J^{g_0} = 0. \end{aligned}$$

Let $B_{g_0}(x,2s)\subset \mathbb {B}\setminus \{0\}$ be a geodesic ball and χ by a cut-off function such that χ=1 in $B_{g_0}(x,s)$. It holds

$$\begin{aligned} \int _{\mathbb {B}} \chi ^2\, \left| d\left( |J^{g_0}|^{\frac{p}{2}} \right) \right| ^2_{g_0}\, d\textrm{vol}_{g_0} \le C\, \int _{\mathbb {B}} |d\chi |^2_{g_0}\, |J^{g_0}|^p\ d\textrm{vol}_{g_0}. \end{aligned}$$

#### Proof

Let δ>0. In order to shorten the notations, we denote $j_{\delta }{:}{=}\left( \delta ^2 + (J^{g_0})^2\right) ^{\frac{p}{4}}$. We have $j_{\delta }>0$ and $j_{\delta }\in C^{\infty }(\Omega _0)$. Moreover, it holds

$$\begin{aligned} \nabla ^{g_0} j_{\delta } = \frac{p}{2}\, \left( \delta ^2 + (J^{g_0})^2 \right) ^{\frac{p}{4}-1}\, J^{g_0}\, \nabla ^{g_0} J^{g_0}. \end{aligned}$$

Since $\Delta _{g_0} J^{g_0}=0$, the Laplacian of $j_{\delta }$ is given by

$$\begin{aligned} \Delta _{g_0} j_{\delta }&= \frac{p}{2}\, \left( \delta ^2 + (J^{g_0})^2 \right) ^{\frac{p}{4}-1}\, \left| \nabla ^{g_0} J^{g_0}\right| ^2_{g_0}\\&\quad + \frac{p}{2}\left( \frac{p}{2}-2\right) \, \left( \delta ^2 + (J^{g_0})^2\right) ^{\frac{p}{4}-2}\, (J^{g_0})^2\, \left| \nabla ^{g_0} J^{g_0}\right| ^2_{g_0} \\&= \frac{p}{2}\, \left( \delta ^2 + (J^{g_0})^2 \right) ^{\frac{p}{4}-2}\, \left| \nabla ^{g_0} J^{g_0}\right| ^2_{g_0} \left[ \delta ^2 + \left( \frac{p}{2}-1\right) \, (J^{g_0})^2 \right] . \end{aligned}$$

Let $B_{g_0}(x,2s)\subset \mathbb {B}\setminus \{0\}$ be a geodesic ball and χ by a cut-off function such that χ=1 in $B_{g_0}(x,s)$. By integration by parts, we obtain

$$\begin{aligned}&\int _{\mathbb {B}} \chi ^2\, |\nabla ^{g_0}j_{\delta }|^2_{g_0}\, d\textrm{vol}_{g_0} \\&\quad = -\int _{\mathbb {B}} \left\langle \nabla ^{g_0}j_{\delta }, 2\chi \, \nabla ^{g_0}\chi \right\rangle _{g_0}\, j_{\delta } + \chi ^2\, j_{\delta }\, (\Delta _{g_0} j_{\delta })\ d\textrm{vol}_{g_0} \\&\quad = -\int _{\mathbb {B}} \left\langle \nabla ^{g_0}j_{\delta }, 2\chi \, \nabla ^{g_0}\chi \right\rangle _{g_0}\, j_{\delta }\\&\qquad + \frac{p}{2}\, \chi ^2\, \left| \nabla ^{g_0} J^{g_0}\right| ^2_{g_0}\, \left( \delta ^2 + (J^{g_0})^2\right) ^{\frac{p}{2}-2} \left[ \delta ^2 + \left( \frac{p}{2}-1\right) \, (J^{g_0})^2 \right] \ d\textrm{vol}_{g_0}. \end{aligned}$$

Hence, we obtain

$$\begin{aligned}&\int _{\mathbb {B}} \chi ^2\, |\nabla ^{g_0}j_{\delta }|^2_{g_0}\, d\textrm{vol}_{g_0} \\&\quad \le \left( \int _{\mathbb {B}} \chi ^2 \left| \nabla ^{g_0} j_{\delta }\right| ^2_{g_0}\, d\textrm{vol}_{g_0}\right) ^{\frac{1}{2}}\, \left( \int _{\mathbb {B}} 4 \left| \nabla ^{g_0}\chi \right| ^2_{g_0}\, j_{\delta }^2\, d\textrm{vol}_{g_0}\right) ^{\frac{1}{2}} \\&\qquad + \frac{p}{2}\int _{\mathbb {B}} \chi ^2\, \left| \nabla ^{g_0} J^{g_0}\right| ^2_{g_0}\, \left( \delta ^2 + (J^{g_0})^2\right) ^{\frac{p}{2}-2} \left[ - \delta ^2 + \left( 1-\frac{p}{2}\right) \, (J^{g_0})^2 \right] \ d\textrm{vol}_{g_0}. \end{aligned}$$

By Young’s inequality, we obtain for any $\theta \in (0,1)$,

20 $$\begin{aligned} \begin{aligned}&(1-\theta )\, \int _{\mathbb {B}} \chi ^2\, |\nabla ^{g_0}j_{\delta }|^2_{g_0}\, d\textrm{vol}_{g_0} \\&\quad \le C(\theta )\, \int _{\mathbb {B}} \left| \nabla ^{g_0}\chi \right| ^2_{g_0}\, j_{\delta }^2\, d\textrm{vol}_{g_0} \\&\qquad + \frac{p}{2}\int _{\mathbb {B}} \chi ^2\, \left| \nabla ^{g_0} J^{g_0}\right| ^2_{g_0}\, \left( \delta ^2 + (J^{g_0})^2\right) ^{\frac{p}{2}-2} \left[ -\delta ^2 + \left( 1-\frac{p}{2}\right) \, (J^{g_0})^2 \right] \ d\textrm{vol}_{g_0}. \end{aligned} \end{aligned}$$

We estimate the last term as follows

$$\begin{aligned}&\int _{\mathbb {B}} \chi ^2\, \left| \nabla ^{g_0} J^{g_0}\right| ^2_{g_0}\, \left( \delta ^2 + (J^{g_0})^2\right) ^{\frac{p}{2}-2} \left[ - \delta ^2 + \left( 1-\frac{p}{2}\right) \, (J^{g_0})^2 \right] \ d\textrm{vol}_{g_0} \\&\quad = -\int _{\mathbb {B}} \chi ^2\, \left| \nabla ^{g_0} J^{g_0}\right| ^2_{g_0}\, \frac{\delta ^2}{ \left( \delta ^2 + (J^{g_0})^2\right) ^{2-\frac{p}{2}} }\ d\textrm{vol}_{g_0} \\&\qquad + \left( 1-\frac{p}{2}\right) \int _{\mathbb {B}} \chi ^2\, \left| \nabla ^{g_0} J^{g_0}\right| ^2_{g_0}\, \left( \delta ^2 + (J^{g_0})^2\right) ^{\frac{p}{2}-2} \, (J^{g_0})^2 \ d\textrm{vol}_{g_0} \\&\quad \le 0 + \frac{4}{p^2}\left( 1-\frac{p}{2}\right) \int _{\mathbb {B}} \chi ^2\, \left| \nabla ^{g_0} j_{\delta }\right| ^2_{g_0}\ d\textrm{vol}_{g_0}. \end{aligned}$$

Coming back to (20), we obtain

$$\begin{aligned} \left( 2-\theta - \frac{2}{p}\right) \, \int _{\mathbb {B}} \chi ^2\, |\nabla ^{g_0}j_{\delta }|^2_{g_0}\, d\textrm{vol}_{g_0}&\le C(\theta )\, \int _{\mathbb {B}} \left| \nabla ^{g_0}\chi \right| ^2_{g_0}\, j_{\delta }^2\, d\textrm{vol}_{g_0} . \end{aligned}$$

Since 1<p≤2, we can choose $\theta =1-\frac{1}{p}$ and we obtain

$$\begin{aligned} \int _{\mathbb {B}} \chi ^2\, |\nabla ^{g_0}j_{\delta }|^2_{g_0}\, d\textrm{vol}_{g_0}&\le C(p)\, \int _{\mathbb {B}} \left| \nabla ^{g_0}\chi \right| ^2_{g_0}\, j_{\delta }^2\, d\textrm{vol}_{g_0} . \end{aligned}$$

We obtain the conclusion by letting $\delta \rightarrow 0$. □

We now show the ε-regularity estimate.

#### Theorem 2.13

Let *g* a metric on $\mathbb {B}\setminus \{0\}$ satisfying the hypothesis of theorem Theorem 2.1, then there exists ε>0 and C>0 depending on the operation ⋆ in (7) and $\gamma _S$ such that the following holds. Assume that

21 $$\begin{aligned} \Vert \textrm{Riem}^g\Vert _{L^2(\mathbb {B},g)} \le \varepsilon . \end{aligned}$$

Then there exists a metric $g_0$ conformal to *g* satisfying

$$\begin{aligned} \Delta _{g_0} J^{g_0} = 0. \end{aligned}$$

Moreover, for each geodesic ball $B_{g_0}(x,s)\subset \mathbb {B}\setminus \{0\}$, it holds

$$\begin{aligned} \Vert \textrm{Sch}^{g_0}\Vert _{L^{2p}(B_{g_0}(x,s))}&\le \ \frac{C}{s^{\frac{2}{p}}}\, \textrm{vol}_{g_0}(B_{g_0}(x,2s))^{\frac{1}{p}-\frac{1}{2}}\, \Vert \textrm{Sch}^{g_0}\Vert _{L^2(B_{g_0}(x,2s))}\\&\quad + C\Vert B^{g_0}\Vert _{L^{\frac{2p}{p+1}}(B_{g_0}(x,2s),g_0)}^{\frac{2}{p+1}} . \end{aligned}$$

#### Remark 2.14

This provides an ε-regularity result for Bach-flat metrics without assuming the scalar curvature to be constant, see for instance [16, 59, 60].

#### Proof

The existence of $g_0$ has been justified at the beginning of Section 2.5. The Schouten tensor satisfies (by (7)):

22 $$\begin{aligned} {\left\{ \begin{array}{ll} \Delta _{g_0}\textrm{Sch}^{g_0} = \textrm{Hess}_{g_0} J^{g_0} + B^{g_0} + \textrm{Riem}^{g_0}\star \textrm{Sch}^{g_0}, \\ \Delta _{g_0} J^{g_0} = 0. \end{array}\right. } \end{aligned}$$

We define $f {:}{=}|\textrm{Sch}^{g_0}|_{g_0}$. This function satisfies

23 $$\begin{aligned} \begin{aligned} -\Delta _{g_0} f&= -\textrm{div}_{g_0}\left( \left\langle \frac{\textrm{Sch}^{g_0}}{|\textrm{Sch}^{g_0}|_{g_0}},\nabla ^{g_0} \textrm{Sch}^{g_0} \right\rangle _{g_0}\right) \\&= -\frac{|\nabla \textrm{Sch}^{g_0}|^2_{g_0}}{|\textrm{Sch}^{g_0}|_{g_0}} + \left\langle \frac{\textrm{Sch}^{g_0}}{|\textrm{Sch}^{g_0}|_{g_0}},-\Delta _{g_0} \textrm{Sch}^{g_0} \right\rangle _{g_0}. \end{aligned} \end{aligned}$$

Hence, for any function $\varphi \in C^{\infty }_c(\mathbb {B};[0,+\infty ))$, we have

$$\begin{aligned}&\int _{\mathbb {B}} \left\langle df,d\varphi \right\rangle _{g_0}\, d\textrm{vol}_{g_0} \\&\quad \le \int _{\mathbb {B}} \varphi \left\langle \frac{\textrm{Sch}^{g_0}}{|\textrm{Sch}^{g_0}|_{g_0}}, - \textrm{Hess}_{g_0} J^{g_0} - B^{g_0} - \textrm{Riem}^{g_0}\star \textrm{Sch}^{g_0} \right\rangle _{g_0}\, d\textrm{vol}_{g_0}. \end{aligned}$$

Let $\chi \in C^{\infty }_c(\mathbb {B}\setminus \{0\};[0,1])$ be a cut-off function such that χ=1 in $B_{g_0}(x,s)$ and χ=0 on $\mathbb {B}\setminus B_{g_0}(x,2s)$ for some s>0 such that $B_{g_0}(x,2s)\subset \mathbb {B}\setminus \{0\}$ and $|d\chi |_{g_0}\le Cs^{-1}$. We consider the test function^3^$\varphi = \chi ^2 f^{p-1}$:

$$\begin{aligned}&\int _{\mathbb {B}} (p-1)\chi ^2 f^{p-2} |df|^2_{g_0} + 2\chi f^{p-1} \left\langle d\chi ,df \right\rangle _{g_0}\, d\textrm{vol}_{g_0} \\&\quad \le \ \int _{\mathbb {B}} \chi ^2\left( f^{p-1} |B^{g_0}|_{g_0} + f^p\, |\textrm{Riem}^{g_0}|_{g_0}\right) \, d\textrm{vol}_{g_0} \\&\qquad - \int _{\mathbb {B}} \chi ^2 f^{p-2}\left\langle \textrm{Sch}^{g_0},\textrm{Hess}_{g_0} J^{g_0} \right\rangle _{g_0}\, d\textrm{vol}_{g_0}. \end{aligned}$$

As a consequence of the above estimate, there exists a constant C>0 depending only on *p* such that the following holds:

24 $$\begin{aligned} \begin{aligned} \int _{\mathbb {B}} \left| d\left( \chi f^{\frac{p}{2}} \right) \right| ^2_{g_0} \, d\textrm{vol}_{g_0}&\le \ C \int _{\mathbb {B}} |d\chi |^2 f^p + \chi ^2\left( f^{p-1} |B^{g_0}|_{g_0} + f^{p} |\textrm{Riem}^{g_0}|_{g_0}\right) \, d\textrm{vol}_{g_0} \\&\quad - \int _{\mathbb {B}} \chi ^2 f^{p-2}\left\langle \textrm{Sch}^{g_0},\textrm{Hess}_{g_0} J^{g_0} \right\rangle _{g_0}\, d\textrm{vol}_{g_0}. \end{aligned} \end{aligned}$$

We now estimate the last term by integration by parts:

$$\begin{aligned}&- \int _{\mathbb {B}} \chi ^2 f^{p-2}\left\langle \textrm{Sch}^{g_0},\textrm{Hess}_{g_0} J^{g_0} \right\rangle _{g_0}\, d\textrm{vol}_{g_0}\\&=\int _{\mathbb {B}} \left\langle \nabla ^{g_0} J^{g_0},\textrm{div}_{g_0}\left( \chi ^2 f^{p-2} \textrm{Sch}^{g_0} \right) \right\rangle _{g_0}\, d\textrm{vol}_{g_0}. \end{aligned}$$

Since $\textrm{div}_{g_0}\textrm{Sch}^{g_0} = \nabla ^{g_0}J^{g_0}$, we obtain the following estimate:

$$\begin{aligned}&- \int _{\mathbb {B}} \chi ^2 f^{p-2}\left\langle \textrm{Sch}^{g_0},\textrm{Hess}_{g_0} J^{g_0} \right\rangle _{g_0}\, d\textrm{vol}_{g_0} \\&\le \ \int _{\mathbb {B}} 2\chi |d\chi |_{g_0} f^{p-1} |dJ^{g_0}|_{g_0} + |p-2|\, \chi ^2 f^{p-2} |df|_{g_0} |dJ^{g_0}|_{g_0} \\&\quad + \chi ^2 f^{p-2} |dJ^{g_0}|_{g_0}^2\, d\textrm{vol}_{g_0}. \end{aligned}$$

By Hölder inequality, we obtain

25 $$\begin{aligned} \begin{aligned}&- \int _{\mathbb {B}} \chi ^2 f^{p-2}\left\langle \textrm{Sch}^{g_0},\textrm{Hess}_{g_0} J^{g_0} \right\rangle _{g_0}\, d\textrm{vol}_{g_0}\\&\quad \le \ 2\left( \int _{\mathbb {B}} |d\chi |^2_{g_0}\ f^p\, d\textrm{vol}_{g_0} \right) ^{\frac{1}{2}} \left( \int _{\mathbb {B}} \chi ^2 f^{p-2} |dJ^{g_0}|_{g_0}^2\, d\textrm{vol}_{g_0} \right) ^{\frac{1}{2}} \\&\qquad +|p-2|\, \left( \int _{\mathbb {B}} \chi ^2 f^{p-2} |df|_{g_0}^2\, d\textrm{vol}_{g_0} \right) ^{\frac{1}{2}} \left( \int _{\mathbb {B}} \chi ^2 f^{p-2} |dJ^{g_0}|^2_{g_0}\, d\textrm{vol}_{g_0} \right) ^{\frac{1}{2}}\\&\qquad + \int _{\mathbb {B}} \chi ^2 f^{p-2} |dJ^{g_0}|_{g_0}^2\, d\textrm{vol}_{g_0}. \end{aligned} \end{aligned}$$

By Young’s inequality, we obtain for any δ>0:

$$\begin{aligned}&- \int _{\mathbb {B}} \chi ^2 f^{p-2}\left\langle \textrm{Sch}^{g_0},\textrm{Hess}_{g_0} J^{g_0} \right\rangle _{g_0}\, d\textrm{vol}_{g_0} \\&\quad \le \ \delta \int _{\mathbb {B}} |d\chi |^2_{g_0}\ f^p\, d\textrm{vol}_{g_0} + \delta \int _{\mathbb {B}} \chi ^2 f^{p-2} |df|_{g_0}^2\, d\textrm{vol}_{g_0}\\&\qquad + C(\delta ) \int _{\mathbb {B}} \chi ^2 f^{p-2} |dJ^{g_0}|_{g_0}^2\, d\textrm{vol}_{g_0}. \end{aligned}$$

Coming back to (24) and using Young inequality, we obtain

$$\begin{aligned} \int _{\mathbb {B}} \left| d\left( \chi f^{\frac{p}{2}} \right) \right| ^2_{g_0} \, d\textrm{vol}_{g_0}&\le C \int _{\mathbb {B}} |d\chi |^2 f^p + \chi ^2\left( f^{p-1} |B^{g_0}|_{g_0} + f^{p} |\textrm{Riem}^{g_0}|_{g_0}\right) \, d\textrm{vol}_{g_0} \\&\quad + C \int _{\mathbb {B}} \chi ^2 f^{p-2} |dJ^{g_0}|^2_{g_0}\, d\textrm{vol}_{g_0}. \end{aligned}$$

Thanks to Lemma 2.10, we obtain

26 $$\begin{aligned} \left( \int _{\mathbb {B}} \chi ^4 f^{2p} \, d\textrm{vol}_{g_0}\right) ^{\frac{1}{2}}&\le C\gamma _S\int _{\mathbb {B}} |d\chi |^2 f^p d\textrm{vol}_{g_0} \end{aligned}$$

27 $$\begin{aligned}&\quad + C\gamma _S\int _{\mathbb {B}} \chi ^2\left( f^{p-1} |B^{g_0}|_{g_0} + f^{p} |\textrm{Riem}^{g_0}|_{g_0}\right) \, d\textrm{vol}_{g_0} \end{aligned}$$

28 $$\begin{aligned}&\quad + C\gamma _S \int _{\mathbb {B}} \chi ^2 f^{p-2} |dJ^{g_0}|^2_{g_0}\, d\textrm{vol}_{g_0}. \end{aligned}$$

We now estimate (27) by Hölder inequality, Lemma 2.9 and using the assumption $\textrm{supp}(\chi )\subset B_{g_0}(x,2s)$:

$$\begin{aligned}&\int _{\mathbb {B}} \chi ^2\left( f^{p-1} |B^{g_0}|_{g_0} + f^{p} |\textrm{Riem}^{g_0}|_{g_0}\right) \, d\textrm{vol}_{g_0} \\&\quad \le \left( \int _{\mathbb {B}} \chi ^{\frac{4p}{p-1}} f^{2p} \right) ^{\frac{p-1}{2p}}\left( \int _{B_{g_0}(x,2s)} |B^{g_0}|_{g_0}^{\frac{2p}{p+1}}\, d\textrm{vol}_{g_0} \right) ^{\frac{p+1}{2p}}\\&\qquad + \left( \int _{\mathbb {B}} \chi ^4 f^{2p} d\textrm{vol}_{g_0} \right) ^{\frac{1}{2}} \left( \int _{B_{g_0}(x,2s)} |\textrm{Riem}^{g_0}|_{g_0}^2\, d\textrm{vol}_{g_0} \right) ^{\frac{1}{2}}. \end{aligned}$$

Thanks to the inequality $\Vert \textrm{Riem}^{g_0}\Vert _{L^2(\mathbb {B},g_0)} \le \Vert \textrm{Riem}^g\Vert _{L^2(\mathbb {B},g)}\le \varepsilon$ by (21) and Remark 2.4, we obtain

$$\begin{aligned}&\int _{\mathbb {B}} \chi ^2\left( f^{p-1} |B^{g_0}|_{g_0} + f^{p} |\textrm{Riem}^{g_0}|_{g_0}\right) \, d\textrm{vol}_{g_0} \\&\quad \le \left( \int _{\mathbb {B}} \chi ^{\frac{4p}{p-1}} f^{2p} \right) ^{\frac{p-1}{2p}}\left( \int _{B_{g_0}(x,2s)} |B^{g_0}|_{g_0}^{\frac{2p}{p+1}}\, d\textrm{vol}_{g_0} \right) ^{\frac{p+1}{2p}} + \varepsilon \left( \int _{\mathbb {B}} \chi ^4 f^{2p} d\textrm{vol}_{g_0} \right) ^{\frac{1}{2}} . \end{aligned}$$

We plug the above estimate in (26), (27) and (28). Using Young inequality, the inequality $\chi ^{\frac{4p}{p-1}}\le \chi ^4$ (since $\chi \le 1$ and $\frac{p}{p-1}\ge 2$ by choice of *p*), we obtain

29 $$\begin{aligned} \left( \int _{\mathbb {B}} \chi ^4 f^{2p} \, d\textrm{vol}_{g_0}\right) ^{\frac{1}{2}} \le&\ C\int _{\mathbb {B}} |d\chi |^2 f^p d\textrm{vol}_{g_0} \end{aligned}$$

30 $$\begin{aligned}&+ C\left( \int _{B_{g_0}(x,2s)} |B^{g_0}|_{g_0}^{\frac{2p}{p+1}}\, d\textrm{vol}_{g_0}\right) ^{\frac{p+1}{p-1}} \end{aligned}$$

31 $$\begin{aligned}&\ + C \int _{\mathbb {B}} \chi ^2 f^{p-2} |dJ^{g_0}|^2_{g_0}\, d\textrm{vol}_{g_0}. \end{aligned}$$

We now use the assumption p≤2 and the inequality $|J^{g_0}|\le f$ to obtain $|J^{g_0}|^{p-2}\ge f^{p-2}$. Using this into (31), we obtain

32 $$\begin{aligned} \int _{\mathbb {B}} \chi ^2 f^{p-2} |dJ^{g_0}|^2_{g_0}\, d\textrm{vol}_{g_0} \le \frac{4}{p^2}\int _{\mathbb {B}} \chi ^2 \left| d\left( (J^{g_0})^{p/2}\right) \right| ^2_{g_0}\, d\textrm{vol}_{g_0}. \end{aligned}$$

Thanks to Lemma 2.12, we have

$$\begin{aligned} \int _{\mathbb {B}} \chi ^2 \left| d\left( (J^{g_0})^{p/2}\right) \right| ^2_{g_0}\, d\textrm{vol}_{g_0} \le C\int _{\mathbb {B}} |d\chi |^2_{g_0} (J^{g_0})^p\, d\textrm{vol}_{g_0} \le C\int _{\mathbb {B}} |d\chi |^2_{g_0} f^p\, d\textrm{vol}_{g_0} . \end{aligned}$$

We plug this inequality into (32) and (31):

$$\begin{aligned} \left( \int _{\mathbb {B}} \chi ^4 f^{2p} \, d\textrm{vol}_{g_0}\right) ^{\frac{1}{2}} \le C\int _{\mathbb {B}} |d\chi |^2 f^p d\textrm{vol}_{g_0} + C\left( \int _{B_{g_0}(x,2s)} |B^{g_0}|_{g_0}^{\frac{2p}{p+1}}\, d\textrm{vol}_{g_0}\right) ^{\frac{p+1}{p-1}}. \end{aligned}$$

Using the inequality $|d\chi |_{g_0}\le Cs^{-1}$ and p≤2, we obtain

$$\begin{aligned} \left( \int _{\mathbb {B}} \chi ^4 f^{2p} \, d\textrm{vol}_{g_0}\right) ^{\frac{1}{2}}&\quad \le \ \frac{C}{s^2}\int _{B_{g_0}(x,2s)} f^p d\textrm{vol}_{g_0} + C\Vert B^{g_0}\Vert _{L^{\frac{2p}{p+1}}(B_{g_0}(x,2s),g_0)}^{\frac{2p}{p-1}} \\&\quad \le \ C\frac{\textrm{vol}_{g_0}(B_{g_0}(x,2s))^{\frac{2-p}{2}}}{s^2}\Vert f\Vert _{L^2(B_{g_0}(x,2s),g_0)}^p\\&\qquad + C\Vert B^{g_0}\Vert _{L^{\frac{2p}{p+1}}(B_{g_0}(x,2s),g_0)}^{\frac{2p}{p-1}} . \end{aligned}$$

□

As a corollary, we obtain an upper bound for the volume of balls thanks to [17]. This is possible thanks to the above ε-regularity.

#### Lemma 2.15

There exist ε>0 and C>0 depending on *p*, the operation ⋆ in (7) and the constants $\gamma _S$ and $\gamma _L$ such that the following holds. Assume that $\Vert \textrm{Riem}^{g}\Vert _{L^2(\mathbb {B},g)} \le \varepsilon$. Then, for each geodesic ball $B_{g_0}(x,s)\subset \mathbb {B}\setminus \{0\}$, we have

$$\begin{aligned} \frac{\textrm{vol}_{g_0}(B_{g_0}(x,s))}{s^4} \le C. \end{aligned}$$

#### Proof

By following the proof of Theorem A in [17] without the renormalization of the metric (in their notation, we have ν=4p), we obtain

33 $$\begin{aligned} \theta (x,s){:}{=}\frac{\textrm{vol}_{g_0}(B_{g_0}(x,s))}{s^4} \le&\ C(p)\left( 1+ s^{(4p-1)\frac{4p-4}{3}} \int _{B_{g_0}(x,s)} |\textrm{Ric}^{g_0}|^{2p}\, d\textrm{vol}_{g_0} \right) . \end{aligned}$$

We also have from [17, page 462] that

$$\begin{aligned}&\textrm{vol}_{g_0}(B_{g_0}(x,2s)) \\&\quad \le C(p)\left( \textrm{vol}_{g_0}(B_{g_0}(x,s)) + s^{4+(4p-1)\frac{4p-4}{3}} \int _{B_{g_0}(x,2s)} |\textrm{Ric}^{g_0}|^{2p}\, d\textrm{vol}_{g_0}\right) . \end{aligned}$$

Dividing by $(2s)^{-4}$, we obtain the following estimate:

34 $$\begin{aligned} \theta (x,2s) \le C\, \theta (x,s) + C\, s^{(4p-1)\frac{4p-4}{3}} \int _{B_{g_0}(x,2s)} |\textrm{Ric}^{g_0}|^{2p}\, d\textrm{vol}_{g_0}. \end{aligned}$$

By Theorem 2.13, we have

$$\begin{aligned} \int _{B_{g_0}(x,s)} |\textrm{Ric}^{g_0}|^{2p}\, d\textrm{vol}_{g_0}&\le \ \frac{C}{s^4}\, \textrm{vol}_{g_0}(B_{g_0}(x,2s))^{2-p}\, \Vert \textrm{Sch}^{g_0}\Vert _{L^2(B_{g_0}(x,2s))}^{2p} \\&\quad + \Vert B^{g_0}\Vert _{L^{\frac{2p}{p+1}}(B_{g_0}(x,2s),g_0)}^{\frac{4p}{p+1}} . \end{aligned}$$

Thus, we obtain

$$\begin{aligned}&s^{(4p-1)\frac{4p-4}{3}} \int _{B_{g_0}(x,s)} |\textrm{Ric}^{g_0}|^{2p}\, d\textrm{vol}_{g_0} \\&\quad \le \ C\, s^{(4p-1)\frac{4p-4}{3}-4}\, \textrm{vol}_{g_0}(B_{g_0}(x,2s))^{2-p}\, \Vert \textrm{Sch}^{g_0}\Vert _{L^2(B_{g_0}(x,2s))}^{2p}\\&\qquad + s^{(4p-1)\frac{4p-4}{3}}\, \Vert B^{g_0}\Vert _{L^{\frac{2p}{p+1}}(\mathbb {B},g_0)}^{\frac{4p}{p+1}} . \end{aligned}$$

Thanks to A.2 and Lemma 2.9 we obtain

$$\begin{aligned}&s^{(4p-1)\frac{4p-4}{3}} \int _{B_{g_0}(x,s)} |\textrm{Ric}^{g_0}|^{2p}\, d\textrm{vol}_{g_0} \\&\quad \le \ C + C\, s^{(4p-1)\frac{4p-4}{3}-4}\, \textrm{vol}_{g_0}(B_{g_0}(x,2s))^{2-p}\, \Vert \textrm{Sch}^{g_0}\Vert _{L^2(B_{g_0}(x,2s))}^{2p} \\&\quad \le C + C\, s^{(4p-1)\frac{4p-4}{3}-4 +4(2-p)}\, \theta (x,2s)^{2-p}\, \Vert \textrm{Sch}^{g_0}\Vert _{L^2(B_{g_0}(x,2s))}^{2p}\\&\quad \le C + C\, s^{(4p-1)\frac{4p-4}{3}+4(1-p)}\, \theta (x,2s)^{2-p}\, \Vert \textrm{Sch}^{g_0}\Vert _{L^2(B_{g_0}(x,2s))}^{2p}. \end{aligned}$$

Using p>1, we obtain the following estimate on the exponent of *s*:

$$\begin{aligned} q{:}{=}(4p-1)\frac{4p-4}{3}+4(1-p) = 4(p-1)\left( \frac{4p-1}{3} - 1 \right) >0. \end{aligned}$$

Therefore, we obtain

35 $$\begin{aligned} s^{(4p-1)\frac{4p-4}{3}} \int _{B_{g_0}(x,s)} |\textrm{Ric}^{g_0}|^{2p}\, d\textrm{vol}_{g_0} \le C+ C\, s^q\, \theta (x,2s)^{2-p}. \end{aligned}$$

If p=2, then Lemma 2.15 is proved. If 1<p<2, then 0<2-p<1. Plugging (35) into (33), we obtain a constant C>0 depending only on *p* such that

36 $$\begin{aligned} \theta (x,s) \le C+ C\, s^q \, \theta (x,2s)^{2-p}. \end{aligned}$$

Plugging (35) into (34), we obtain

37 $$\begin{aligned} \theta (x,2s) \le C\, \theta (x,s) + C\, s^q\, \theta (x,2s)^{2-p}. \end{aligned}$$

Combining (36) and (37), we obtain

$$\begin{aligned} \theta (x,2s) \le C + C\, s^q\, \theta (x,2s)^{2-p}. \end{aligned}$$

Since 2-p<1, we apply Young’s inequality and obtain Lemma 2.15. □

### Singularity Removability

In this section, we prove that $g_0$ extends across the origin, up to reparametrization.

#### Lemma 2.16

There exists a diffeomorphism Ψ of $\mathbb {B}\setminus \{0\}$ such that $\Psi ^*g_0\in W^{2,2p}(\mathbb {B})$.

To prove Lemma 2.16, we proceed to a blow-up around the origin and prove that the strong limit in the $W^{2,2p}_{\textrm{loc}}$-topology is a flat space. Thus, we can extend our metric before the origin. For each $s\in (0,\frac{1}{8})$, we consider the metric $h_s {:}{=}s^{-2} g_0(s\cdot )$ on $\mathbb {B}_{1/s}\setminus \{0\}$. The metric $h_s$ still satisfies the following property.

#### Lemma 2.17

There exist ε>0 and C>0 depending on *p*, and the constants appearing in the assumptions of Theorem 2.1 such that the following holds. Assume that $\Vert \textrm{Riem}^g\Vert _{L^2(\mathbb {B},g)}\le \varepsilon$. Then, for each geodesic ball $B_{h_s}(x,1)\subset \mathbb {B}_{1/s}\setminus \{0\}$, it holds

$$\begin{aligned} \Vert \textrm{Sch}^{h_s}\Vert _{L^{2p}(B_{h_s}(x,1))} \xrightarrow [s\rightarrow 0]{} 0. \end{aligned}$$

#### Proof

We have the following relations:

$$\begin{aligned}&s^2 \textrm{Riem}^{h_s} = \textrm{Riem}^{g_0}, \quad s^4 d\textrm{vol}_{h_s} = d\textrm{vol}_{g_0},\quad s^{-2} B^{h_s} = B^{g_0},\\&\textrm{Sch}^{h_s} =\textrm{Sch}^{g_0}, \quad s^{-2} J^{h_s} = J^{g_0}, \quad B_{h_s}(x,1) = B_{g_0}(x,s),\\&|\textrm{Sch}^{g_0}|^2_{g_0} = s^{-4}|\textrm{Sch}^{h_s}|^2_{h_s},\quad |B^{g_0}|_{g_0} = s^{-4}|B^{h_s}|_{h_s}. \end{aligned}$$

Fix a ball $B_{h_s}(x,1)\subset \mathbb {B}_{1/s}\setminus \{0\}$ and let $\theta {:}{=}\frac{1}{4}\textrm{dist}_{\textrm{eucl}}(x,\{0\})$. By Theorem 2.13, we have the following estimate:

$$\begin{aligned} \Vert \textrm{Sch}^{g_0}\Vert _{L^{2p}(B_{g_0}(sx,s\theta ))}&\le \frac{C}{s^{\frac{2}{p}}}\textrm{vol}_{g_0}(B_{g_0}(sx,2s\theta ))^{\frac{1}{p}-\frac{1}{2}}\Vert \textrm{Sch}^{g_0}\Vert _{L^2(B_{g_0}(sx,2s\theta ))} \\&\quad +C \Vert B^{g_0}\Vert _{L^{\frac{2p}{p+1}}(\mathbb {B},g_0)}^{\frac{2}{p+1}} . \end{aligned}$$

We multiply by $s^{2-\frac{2}{p}}$ in order to obtain a scale-invariant formulation of the left-hand side:

$$\begin{aligned}&\left( s^{4p-4}\int _{B_{g_0}(sx,s\theta )} |\textrm{Sch}^{g_0}|^{2p}\, d\textrm{vol}_{g_0} \right) ^{\frac{1}{2p}} \\&\quad \le C\left( \frac{ \textrm{vol}_{g_0}(B_{g_0}(sx,2s\theta )) }{s^4} \right) ^{\frac{1}{p}-\frac{1}{2}} \Vert \textrm{Sch}^{g_0}\Vert _{L^2(B_{g_0}(sx,2s\theta ))} + Cs^{2-\frac{2}{p}} \Vert B^{g_0}\Vert _{L^{\frac{2p}{p+1}}(\mathbb {B},g_0)}^{\frac{2}{p+1}} . \end{aligned}$$

By Lemma 2.15, we obtain

$$\begin{aligned} \left( s^{4p-4}\int _{B_{g_0}(sx,s\theta )} |\textrm{Sch}^{g_0}|^{2p}\, d\textrm{vol}_{g_0} \right) ^{\frac{1}{2p}}&\le \ C \Vert \textrm{Sch}^{g_0}\Vert _{L^2(B_{g_0}(sx,2s\theta ))} \\&\quad + Cs^{2-\frac{2}{p}} \Vert B^{g_0}\Vert _{L^{\frac{2p}{p+1}}(\mathbb {B},g_0)}^{\frac{2}{p+1}} . \end{aligned}$$

Here, the constant *C* depends on θ, by Lemma 2.15. Since p>1, it holds $2-\frac{2}{p}>0$ and we obtain

$$\begin{aligned} \Vert \textrm{Sch}^{h_s}\Vert _{L^{2p}(B_{h_s}(x,1))} = \left( s^{4p-4}\int _{B_{g_0}(sx,s\theta )} |\textrm{Sch}^{g_0}|^{2p}\, d\textrm{vol}_{g_0} \right) ^{\frac{1}{2p}} \xrightarrow [s\rightarrow 0]{} 0. \end{aligned}$$

□

Thanks to [65, Theorem 7.1] (see also [48, Theorem 4.2] and [30, 39, 53, 64]) together with Lemma 2.17, for any ball $B_{h_s}(x,t)$, there exist harmonic coordinates $(x^1,\ldots ,x^4)$ such that the components $(h_s)_{ij} \in W^{2,2p}(B_{\xi }(x,t/2))$ satisfy the following properties

$$\begin{aligned}&\Vert x^i\Vert _{L^{\infty }(B_{\xi }(x,t/2))} \le 2t,\\&\Vert \nabla x^i\otimes \nabla x^j - \xi ^{ij} \Vert _{L^{\infty }(B_{\xi }(x,t/2))}\le C\left( t^{4p-4} \int _{B_{h_s}(x,t)} |\textrm{Sch}^{h_s}|^{2p}_{h_s}\, d\textrm{vol}_{h_s} \right) ^{\frac{1}{2p}},\\&\Vert \nabla ^2 (h_s)_{ij} \Vert _{L^{2p}(B_{\xi }(x,t/2))} \le Ct^{-2\frac{p-1}{p}} \Vert \textrm{Sch}^{h_s}\Vert _{L^{2p}(B_{h_s}(x,t))}. \end{aligned}$$

Up to a subsequence, the metrics $(h_s)_{s>0}$ converge as s→0 in the weak $W^{2,2p}_{\textrm{loc}}$-topology to some metric $h_0$ on $\mathbb {R}^4\setminus \{0\}$. In particular, we deduce that $(\textrm{Riem}^{h_s})_{s>0}$ converges to $\textrm{Riem}^{h_0}$ weakly in $L^{2p}_{\textrm{loc}}$. From the following relation, we obtain that $\textrm{Riem}^{h_0}=0$:

$$\begin{aligned} \Vert \textrm{Riem}^{h_s}\Vert _{L^2\left( B_{h_s}(x,t),h_s\right) } = \Vert \textrm{Riem}^{g_0}\Vert _{L^2\left( B_{g_0}(sx,st),g_0 \right) } \xrightarrow [s\rightarrow 0]{} 0. \end{aligned}$$

In the spirit of the singularity removability for Ricci-flat metrics, see for instance [31, 56, 57], we have the following result.

#### Claim 2.18

There exists a diffeomorphism $\Phi :\mathbb {R}^4\setminus \{*\} \rightarrow \mathbb {R}^4\setminus \{0\}$ such that $\Phi ^*h_0 = \xi$.

#### Remark 2.19

Here, we strongly use the fact that we are in dimension greater than 2, so that $\mathbb {R}^4\setminus \{0\}$ is simply connected.

#### Proof

Since $\textrm{Riem}^{h_0}=0$, for every $x\in \mathbb {R}^4\setminus \{0\}$, there exists an open set $U_x\subset \mathbb {R}^4\setminus \{0\}$ containing *x*, another open set $V_x\subset \mathbb {R}^4$ and an isometry $\phi _x :U_x\rightarrow V_x$. If $y\in \mathbb {R}^4\setminus \{0,x\}$ is such that $U_x\cap U_y\ne \emptyset$, then $\phi _x\circ \phi _y^{-1}:\phi _y(U_y\cap U_x) \rightarrow \phi _x(U_y\cap U_x)$ is an isometry of $\mathbb {R}^4$. Thus, $\phi _x\circ \phi _y^{-1}$ is the composition of some rotations and translations. Up to changing $\phi _y$ by an isometry of $\mathbb {R}^4$, we can assume that $\phi _x\circ \phi _y^{-1} = \textrm{id}$.

Therefore, given any $x\in \mathbb {R}^4\setminus \{0\}$ and any path $\gamma :[0,1]\rightarrow \mathbb {R}^4\setminus \{0\}$ such that γ(0)=x, the map $\phi _x$ can be analytically continued along γ, in the sense of [46, Corollary 12.3]. Given $y\in \mathbb {R}^4\setminus \{0,x\}$, we define Φ(y) to be the value at *y* of any analytic continuation of $\phi _x$ from *x* to *y*. This is well defined, since *X* is simply connected, thanks to [46, Theorem 12.2]. Then, the map $\Phi :(\mathbb {R}^4\setminus \{0\},h_0)\rightarrow (\mathbb {R}^4,\xi )$ is a local isometry.

Consider now $(X_n)_{n\in \mathbb {N}}$ an exhaustion of $\mathbb {R}^4\setminus \{0\}$ by simply connected compact sets, *i.e.* each $X_n$ is compact in $\mathbb {R}^4\setminus \{0\}$, if n≤m then $X_n \subset X_m$ and $\mathbb {R}^4\setminus \{0\} = \bigcup _{n\in \mathbb {N}} X_n$. Since each $X_n$ is complete, we obtain that $\Phi :X_n\rightarrow \Phi (X_n)$ is a Riemannian covering map thanks to [46, Theorem 6.3]. Applying [46, Corollary A.59], we deduce that each $\Phi :X_n\rightarrow \Phi (X_n)$ is a diffeomorphism. Thus $\Phi :X_n\rightarrow \Phi (X_n)$ is a global isometry (a local isometry preserves the arc-length, so that a bijective local isometry is a global isometry). This is valid for any *n*. Hence $\Phi :\mathbb {R}^4\setminus \{0\}\rightarrow \Phi (\mathbb {R}^4\setminus \{0\})$ is a bijective global isometry. Thus $\Phi (\mathbb {R}^4\setminus \{0\})$ has the same topology as $\mathbb {R}^4\setminus \{0\}$ and we obtain that the map $\Phi :(\mathbb {R}^4\setminus \{0\},h_0)\rightarrow (\mathbb {R}^4\setminus \{*\},\xi )$ is a global isometry. □

Consequently, $(\Phi ^*h_s)_{s>0}$ has only one adherence value as s→0, meaning that $\Phi ^* h_s\xrightarrow [s\rightarrow 0]{} \xi$ in the weak $W^{2,2p}_{\textrm{loc}}$-topology. In other words there exists a diffeomorphism $\Psi :\mathbb {B}\setminus \{0\}\rightarrow \mathbb {B}\setminus \{0\}$ such that $\Psi ^* g_0$ converges to ξ at the origin in the weak $W^{2,2p}$-topology. Hence, $\Psi ^* g_0\in W^{2,2p}(\mathbb {B})\hookrightarrow C^0(\mathbb {B})$. This proves Lemma 2.16.

## Asymptotically Flat and Asymptotically Locally Euclidean Manifolds

In this section we are interested in non-compact Riemannian manifolds with a single end, which are asymptotically locally Euclidean. By this term, we mean the following definition:

### Definition 3.1

Let $k\in \mathbb {N}$ and $\alpha \in (0,1)$. A Riemannian manifold (*M*, *g*), non-compact with a single end, is said to be $C^{k,\alpha }$-asymptotically locally Euclidean (ALE) of order τ>0 if there exist a compact subset K⊂M, R>0 and Γ is a finite group of *SO*(*n*) acting freely and a $C^{k+1,\alpha }$-diffeomorphism (coordinate chart at infinity)

$$ \Phi :M \setminus K \rightarrow (\mathbb {R}^n \setminus B(0, R)) / \Gamma $$

such that the components of the metric in this chart, $g_{ij}$, satisfy:

$$\begin{aligned} {\left\{ \begin{array}{ll} g_{ij}(z) = \delta _{ij} + O(|z|^{-\tau }),\\ \partial ^m g_{ij}(z) = O(|z|^{-\tau -m}), \quad \forall m \in \{1, \dots , k\},\\ {[}\partial ^k g_{ij}]_\alpha (z) = O(|z|^{-\tau -k-\alpha }), \end{array}\right. } \end{aligned}$$

where

$$ [\partial ^k g_{ij}]_\alpha (z) = \sup _{0<d_g(z,z')\le 1} \frac{|\partial ^k g_{ij}(z) - \partial ^k g_{ij}(z')|}{|z - z'|^\alpha }. $$

For brevity, we write: $g - \delta \in C^{k, \alpha }_{-\tau }(M)$. If $\Gamma = \{1\}$ then (*M*, *g*) is said to be $C^{k,\alpha }$-asymptotically flat of order τ.

In Theorem 1.6, Herzlich proved that a necessary condition for an ALE manifold (*M*, *g*) to have a $C^{0,\delta }$-compactification is the existence of some $1> \alpha \ge \eta >0$ such that $W^g\in C^{0,\alpha }_{-2-\eta }(M)$ and $\textrm{Cot}^g\in C^{0,\alpha }_{-3-\eta }(M)$. Differentiating once more, a similar assumption concerning the Bach tensor would be $B^g\in C^{0,\alpha }_{-4-\eta }(M)$, which implies $B^g\in L^p$ for some p>1. In our case, we consider a metric *g* such that $g-\delta \in C^{1}_\tau (M)$ for some τ>0. Then we make the naive compactification, considering $h{:}{=}\vert x\vert ^4 \iota ^*g$ where $\iota :z\mapsto \frac{z}{|z|^2}$ is the inversion. Hence, we have the following asymptotic expansion:

$$ h_{ij} \underset{r\rightarrow 0}{=} \delta _{ij} + O\left( r^\tau \right) , \qquad \det h \underset{r\rightarrow 0}{=} 1+o(1)\qquad \text { and } \qquad \Gamma _h \underset{r\rightarrow 0}{=}O\left( r^\tau \right) , $$

where $\Gamma _h$ are the Christoffel symbol of *h*. Thanks to those estimates, we can easily check that the assumptions 1 and 3 to 6 of Theorem 1.7 are satisfied.

We now check the assumption $\textrm{Riem}^{h}\in L^2(\mathbb {B},h)$. First, since the Weyl tensor is conformally invariant, we have

$$\begin{aligned} | W^{h} |_{h}^2\, \sqrt{\det h}\in L^1. \end{aligned}$$

Let $\tilde{h} {:}{=}\iota ^*g$, then the Schouten tensor changes according to the formula (3):

$$\begin{aligned} |\textrm{Sch}^h|_{h}&\le |\textrm{Sch}^{\tilde{h}}|_{h} + \left| \textrm{Hess}_{\tilde{h}}(2\log r) - d(2\log r)\otimes d(2\log r)+ \frac{|d(2\log r)|^2_{\tilde{h}}}{2} {\tilde{h}} \right| _{h} \\&\le r^{-4}|\textrm{Sch}^{\tilde{h}}|_{\tilde{h}} + \left| \textrm{Hess}_{\tilde{h}}(2\log r) - d(2\log r)\otimes d(2\log r)+ \frac{|d(2\log r)|^2_{\tilde{h}}}{2} \tilde{h} \right| _{h}. \end{aligned}$$

The Hessian changes according to the following formula, see for instance [25, Equation (2)]: if $\tilde{h}=e^{2u} h$ and $f\in C^{\infty }$, then

$$\begin{aligned} (\textrm{Hess}_{\tilde{h}} f)_{ij}&= \nabla ^{\tilde{h}}_i \nabla ^{\tilde{h}}_j f \\&= \nabla ^{h}_i \nabla ^{\tilde{h}}_j f - (\nabla ^h_i u) (\nabla ^{\tilde{h}}_j f) - (\nabla ^h_j u) (\nabla ^{\tilde{h}}_i f) + \left\langle \nabla ^h u, \nabla ^{\tilde{h}} f \right\rangle _h h_{ij} \\&= (\textrm{Hess}_h f)_{ij} -2(\nabla ^h u\otimes \nabla ^h f)_{ij} + \left\langle du,df \right\rangle _h h_{ij} . \end{aligned}$$

We apply this formula to the choice $\tilde{h}=e^{2u} h$ with u=-2logr and f=2logr:

$$\begin{aligned}&\textrm{Hess}_ {\tilde{h}}(2\log r) - d(2\log r)\otimes d(2\log r)+ \frac{|d(2\log r)|^2_{ \tilde{h}}}{2} \tilde{h} \\&\quad = \textrm{Hess}_{h}(2\log r) +d(2\log r)\otimes d(2\log r) - \frac{|d(2\log r)|^2_{h}}{2} h. \end{aligned}$$

The first order of the above expression in the expansion as r→0 is obtained by changing *h* with the Euclidean metric:

$$\begin{aligned} -\frac{2}{r^2}dr\otimes dr +\frac{4}{r^2}dr\otimes dr - \frac{2}{r^2} (dr^2 +r^2d\theta ^2) = -2\, d\theta ^2. \end{aligned}$$

Hence, we obtain

$$\begin{aligned} \int _{\mathbb {B}} |\textrm{Sch}^{h}|^2_{h}\, d\textrm{vol}_{h} \le C\int _{\mathbb {B}} |\textrm{Sch}^{ \tilde{h}}|^2_{ \tilde{h}}\, d\textrm{vol}_{ \tilde{h}} + C. \end{aligned}$$

Finally the Bach tensor satisfies $\vert B^h\vert _h = \vert x\vert ^{-8} \vert B^{\tilde{h}}\vert _{\tilde{h}}$, hence we easily check that $\vert B^h\vert _h \in L^{p'}$ for $1<p'<\frac{3p}{1+2p}$. Thus, the metric *h* can be conformally extended in the $W^{2,2p'}$-topology across the origin and Corollary 1.8 is proved.

## Isolated Singularities of $W^{4,\frac{4}{3}+\varepsilon }$-Immersions

In this section, we apply Theorem 2.1 to immersions whose second fundamental form is twice differentiable.

### Theorem 4.1

Let $\varepsilon \in (0,\frac{1}{10})$ and d≥5 be an integer. There exists δ>0 depending only on *d* and ε such that the following holds. Consider $\Phi :\mathbb {B}^4\setminus \{0\}\rightarrow \mathbb {R}^d$ be a smooth immersion such that its second fundamental form $\mathrm {I\!I}_{\Phi }$ satisfies

$$\begin{aligned} \int _{\mathbb {B}} |\nabla ^2\mathrm {I\!I}_{\Phi }|^{\frac{4}{3}+\varepsilon }_{g_{\Phi }} + |\nabla \mathrm {I\!I}|^{2+\varepsilon }_{g_{\Phi }} + |\mathrm {I\!I}|^{4+\varepsilon }_{g_{\Phi }}+1\, d\textrm{vol}_{g_{\Phi }} <+\infty . \end{aligned}$$

Assume that

$$\begin{aligned} \int _{\mathbb {B}} |\mathrm {I\!I}|^4_{g_{\Phi }}\, d\textrm{vol}_{g_{\Phi }} <\delta . \end{aligned}$$

Then, there exist a diffeomorphism $f\in \textrm{Diff}(\mathbb {B}^4\setminus \{0\})$, a conformal factor $u\in C^{\infty }(\mathbb {B}^4\setminus \{0\})$, an number p>2 and a metric *h* on $\mathbb {B}^4$ of class $W^{2,p}(\mathbb {B}^4)$ such that $g_{\Phi \circ f} = e^{2u} h$.

### Proof

By Gauss–Codazzi equations, we have

$$\begin{aligned} \textrm{Riem}^{g_{\Phi }}_{ijkl} = \left\langle \mathrm {I\!I}_{ik},\mathrm {I\!I}_{jl} \right\rangle - \left\langle \mathrm {I\!I}_{il},\mathrm {I\!I}_{jk} \right\rangle \in L^2(\mathbb {B},g_{\Phi }). \end{aligned}$$

We deduce that the Bach tensor of $g_{\Phi }$ is also quadratic in $\mathrm {I\!I}$:

$$\begin{aligned} |B^{g_{\Phi }}|_{g_{\Phi }} \le C \left( |\nabla ^2\mathrm {I\!I}|_{g_{\Phi }} |\mathrm {I\!I}|_{g_{\Phi }} + |\nabla \mathrm {I\!I}|_{g_{\Phi }}^2 + |\mathrm {I\!I}|_{g_{\Phi }}^4 \right) . \end{aligned}$$

Thus, we obtain $B^{g_{\Phi }}\in L^p(\mathbb {B},g_{\Phi })$ for some p=p(ε)>1. Therefore, the assumptions A.1 and A.2 of Theorem 2.1 are satisfied. The Sobolev inequality A.3 is verified in (2). The assumption A.4 follows from a uniform estimate of the Green function for Dirichlet boundary conditions. As explained in [15], this is a consequence of the Sobolev inequality. □

## Footnotes

1 If we consider the case where *g* is invariant by the action of a finite group of $\textrm{SO}\left( 4,\mathbb {R} \right)$, we can also consider the case of orbifolds.

2 In other words, the pull-back of the metric *g* is $C^{\infty }$ on $\mathbb {B}^4\setminus \{0\}$, and does not a priori extends smoothly to $\mathbb {B}^4$, which would provide a smooth orbifold metric.

3 Proceeding exactly as in Lemma 2.12 and using (23) with the fact that $\textrm{Sch}^{g_0}$ and $\Delta _{g_0}\textrm{Sch}^{g_0}$ lie in $L^{\infty }_{\textrm{loc}}(\mathbb {B}\setminus \{0\})$, we have that $f^{\frac{p}{2}}\in W^{1,2}_{\textrm{loc}}(\mathbb {B}\setminus \{0\})$, so that all the quantities involved are well-defined.
